# Recent progress in self‐powered multifunctional e‐skin for advanced applications

**DOI:** 10.1002/EXP.20210112

**Published:** 2022-01-14

**Authors:** Yunfeng Chen, Zhengqiu Gao, Fangjia Zhang, Zhen Wen, Xuhui Sun

**Affiliations:** ^1^ Institute of Functional Nano and Soft Materials (FUNSOM), Jiangsu Key Laboratory for Carbon‐Based Functional Materials and Devices Soochow University Suzhou P. R. China

**Keywords:** coupling effects, electronic skin, self‐powered sensor, single effect

## Abstract

Electronic skin (e‐skin), new generation of flexible wearable electronic devices, has characteristics including flexibility, thinness, biocompatibility with broad application prospects, and a crucial place in future wearable electronics. With the increasing demand for wearable sensor systems, the realization of multifunctional e‐skin with low power consumption or even autonomous energy is urgently needed. The latest progress of multifunctional self‐powered e‐skin for applications in physiological health, human–machine interaction (HMI), virtual reality (VR), and artificial intelligence (AI) is presented here. Various energy conversion effects for the driving energy problem of multifunctional e‐skin are summarized. An overview of various types of self‐powered e‐skins, including single‐effect e‐skins and multifunctional coupling‐effects e‐skin systems is provided, where the aspects of material preparation, device assembly, and output signal analysis of the self‐powered multifunctional e‐skin are described. In the end, the existing problems and prospects in this field are also discussed.

## Graphical abstract

In this review, we compile the latest work of self‐powered multifunction e‐skin, discussing related energy sources and coupling techniques. In addition, the latest progress of multifunctional self‐powered e‐skin for applications in physiological health, human–machine interaction (HMI), virtual reality (VR), and artificial intelligence (AI) are also present.

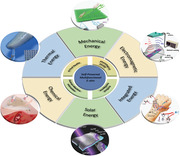

## 1 INTRODUCTION

E‐skin is a novel type of flexible wearable sensor, which is as transparent, soft, and thin as skin.^[^ ^1^, ^2^, ^3^ ^]^ By attaching e‐skin to the robot's fingers and arms, the robot can obtain the ability to feel an external touch like human skin.^[^ ^4^, ^5^, ^6^ ^]^ Inspired by human skin, many efforts have been devoted to regenerating the tactile sensory function,^[^ ^7^, ^8^, ^9^, ^10^, ^11^, ^12^ ^]^ but e‐skin not only needs to acquire haptic perception, but more importantly, it can distinguish between multiple stimuli at the same time, thereby further gaining multidimensional perception. E‐skin is the core of the future network of wearable electronic devices, with characteristics including multifunction, ultra‐thin, low and even zero power consumption, good flexibility, and biocompatibility which makes a lot of sense. For multifunctional e‐skin, different types of sensing units (e.g., pressure, humidity, and temperature sensors) are integrated on a flexible substrate.^[^ ^13^, ^14^, ^15^ ^]^

However, the integration of multiple sensors and complex signal transmission modules significantly increases the energy consumption of the whole e‐skin system. Although current power units such as flexible batteries and flexible supercapacitors have made great progress, they still bring a series of restrictions, such as limiting the ultra‐thinness and flexibility of e‐skin, requiring frequent charging, and battery replacement.^[^ ^16^, ^17^, ^18^, ^19^, ^20^, ^21^, ^22^, ^23^ ^]^ With the future development of wearable devices, the realization of multifunctional e‐skin with low power consumption and even autonomous energy is the key to realize the next generation of portable and wearable e‐skin in various applications.^[^ ^24^, ^25^, ^26^, ^27^, ^28^ ^]^

**FIGURE 1:**
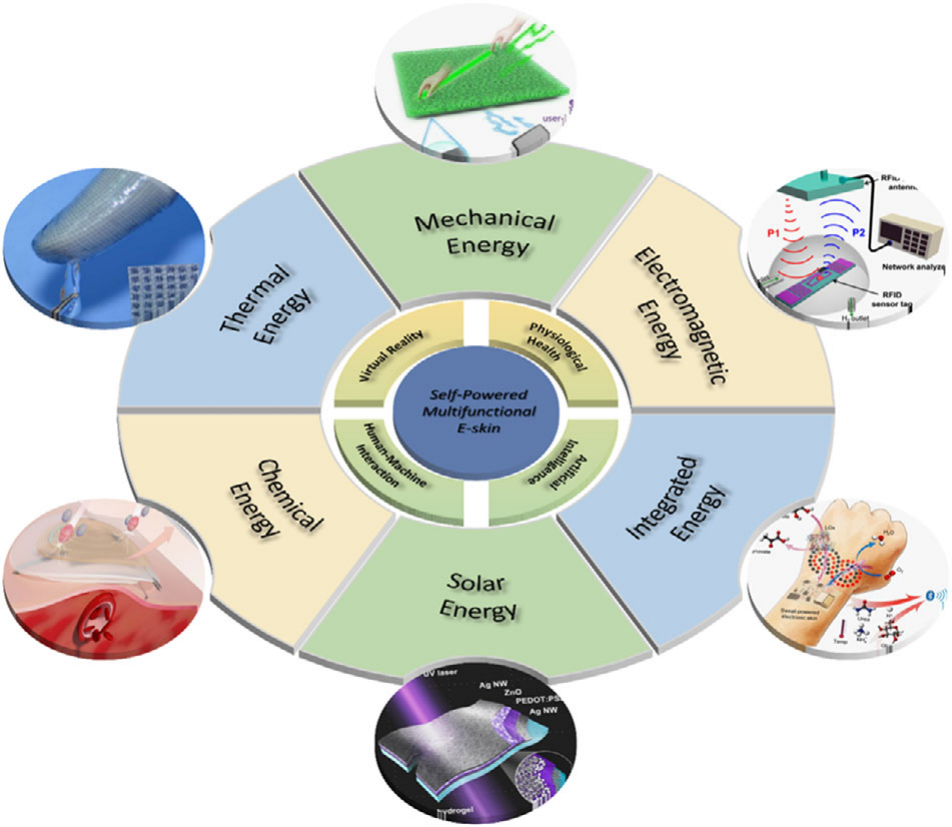
Relationship between different energy sources and applications of self‐powered multifunctional e‐skin. Upper left: Reproduced with permission.^[^ ^78^ ^]^ Copyright 2015, Springer Nature. Up: Reproduced with permission.^[^ ^106^ ^]^ Copyright 2020, American Association for the Advancement of Science. Upper right: Reproduced with permission.^[^ ^74^ ^]^ Copyright 2015, American Chemical Society. Bottom right: Reproduced with permission.^[^ ^87^ ^]^ Copyright 2020, American Association for the Advancement of Science. Down: Reproduced with permission.^[^ ^67^ ^]^ Copyright 2020, American Chemical Society. Bottom left: Reproduced with permission.^[^ ^85^ ^]^ Copyright 2019, American Chemical Society

In this review, we summarize the latest development of self‐powered multifunctional e‐skin, combining its application prospects in the fields of physiological health, human–machine interaction (HMI), virtual reality (VR), and artificial intelligence (AI). As is demonstrated in Figure 1, the classification discusses the realization of self‐powered energy sources, that is, self‐powering through harvesting mechanical energy, solar energy, chemical energy, electromagnetic energy. It is worth noting that the “self‐powered” mentioned here has two main meanings, one is that the system achieves active zero‐power sensing through the energy generated by itself, that is, the generation of sensing signal does not consume external energy. Another refers to the latter's measurement circuit and signal transmission, which also enables fully autonomous e‐skin systems. What's more, we summarize a variety of self‐powered effects coupled with e‐skin, as well as their corresponding multifunctional characteristics (e.g., temperature & pressure, pressure & humidity, temperature & pressure & optics). Finally, we have a prospective summary toward the development and problems of self‐powered multifunctional e‐skin.

## 2 SOURCE OF ELECTRICAL ENERGY

So far, numerous studies about self‐powered e‐skin have been conducted to detect signals from the environment, such as photodetector for testing light intensity based on photovoltaic effect,^[^ ^29^, ^30^, ^31^, ^32^ ^]^ the temperature sensor for the perception of surrounding temperature based on thermoelectric effect,^[^ ^33^, ^34^, ^35^, ^36^ ^]^ biofuel cells based on the chemical energy,^[^ ^37^, ^38^, ^39^, ^40^ ^]^ or wireless sensor system based on RFID^[^ ^41^, ^42^ ^]^ and piezoelectric nanogenerator (PENG)^[^ ^43^, ^44^, ^45^, ^46^, ^47^, ^48^ ^]^ and triboelectric nanogenerator (TENG)^[^ ^49^, ^50^, ^51^, ^52^, ^53^, ^54^ ^]^ for the perception of mechanical energy based on the piezoelectric and triboelectric effect. In this section, the basic mechanisms of the most promising self‐powered multifunctional e‐skin will be compiled and discussed in detail.

### 2.1 Mechanical energy

Mechanical energy, as a ubiquitous energy source, is one of the most common and easily accessible energies in our lives. The frequencies of available mechanical vibration in the environment range from a few Hz (human steps, human heart rate, and the waves of the sea) to a few kHz (the mechanical engine).^[^ ^55^, ^56^ ^]^ To meet the needs of different frequencies and pressure regions for self‐powered e‐skin, the piezoelectric effect and triboelectric effect have come into being.

#### Piezoelectric effect

**FIGURE 2:**
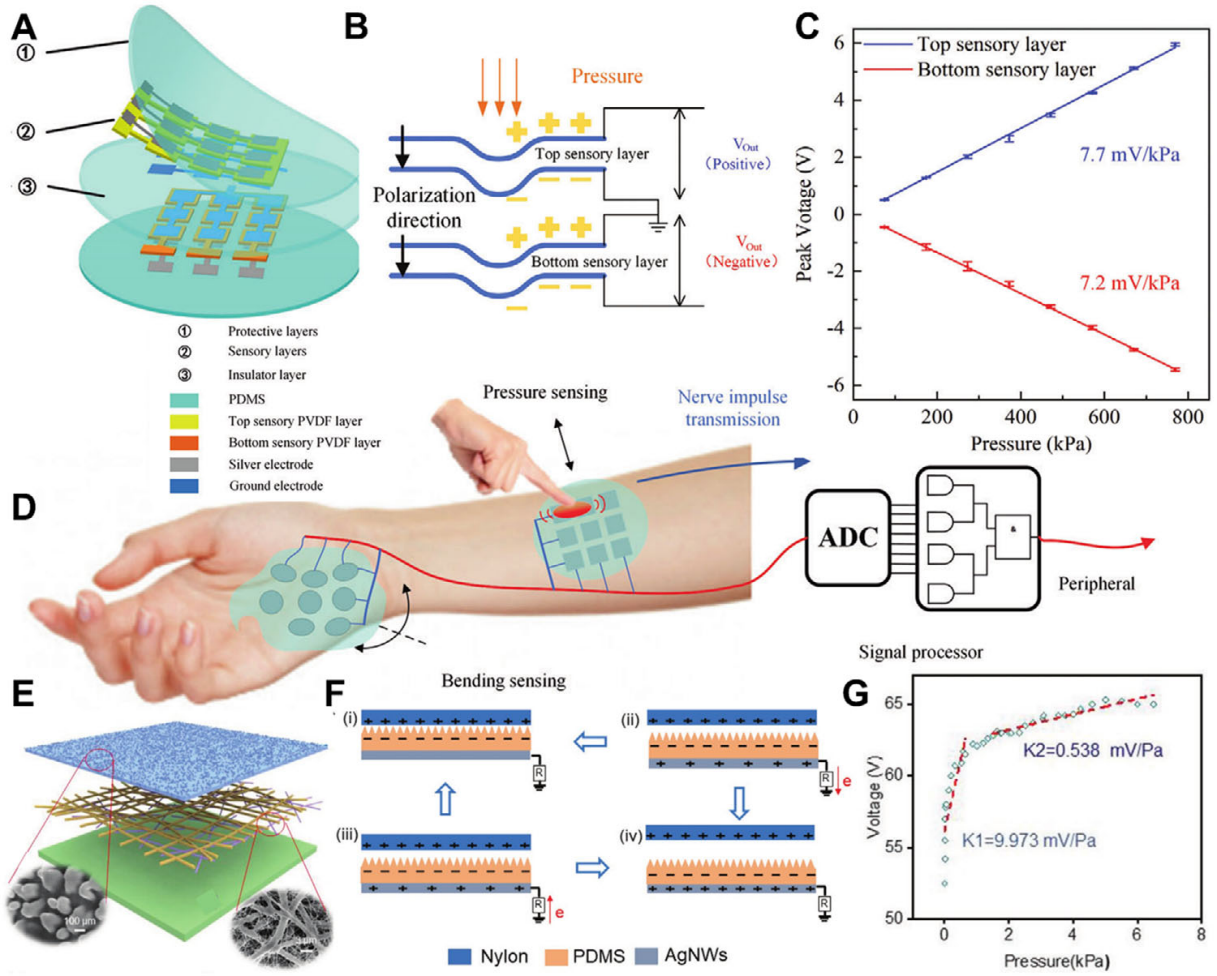
Response of electronic skin to mechanical energy. (A) Illustration of the piezoelectric tactile sensor array. (B) Schematic of piezoelectric sensory films. (C) Output peak voltage versus normal force from 80 to 750 kPa. (D) Schematic of mechanical signal detection by the sensor array. Reproduced with permission.^[^ ^61^ ^]^ Copyright 2021, Wiley‐VCH. (E) The detailed structure of the TENG. (F) Schematics of the operating principle for the TENG. (G) Output voltage of the TENG against applied pressure. Reproduced with permission.^[^ ^65^ ^]^ Copyright 2020, Wiley‐VCH

Piezoelectricity results from the deformation of an external object on a piezoelectric material. The first PENG, developed by Wang and Song in 2006, is based on the **piezoelectric** effect of ZnO nanowire and can convert the collected mechanical energy into piezoelectricity.^[^ ^57^ ^]^ On this basis, several self‐powered e‐skin based on the piezoelectric effect has been prepared to accurately identify the pressure applied to the e‐skin.^[^ ^58^, ^59^, ^60^ ^]^ Recently, many studies have explored advanced structures including nanostructures and two‐dimensional (2D) materials.^[^ ^61^ ^]^ Mimicking human skins, Lin et al. use two protective layers, two sensory layers, and one insulative layer to design the tactile sensor array (Figure 2A). The fundamental mechanism of e‐skin based on the piezoelectric effect could be clearly understood from Figure 2B, the applied pressure causes a change in the dipoles' separation in the material, resulting in a buildup of electric charges that occurred on the electrodes. According to Figure 2C, under the bending stimuli mode, the output voltage of the two sensory layers will be kept synchronized and the voltage signal relates to the bending radius and angle. Attached to the human body, this special structure of piezoelectric e‐skin can easily simulate the biological process of human beings, as depicted in Figure 2D.

#### Triboelectric effect

As another promising energy conversion device reported by Fan et al. in 2012 for personal wearable electronics that could autonomously harvest mechanical energy,^[^ ^62^ ^]^ TENGs have attracted particular interest in recent years. For instance, pressure‐sensing e‐skin based on TENG realizes passive‐active sensing by converting its mechanical stimuli into electrical signals. By using contact electrification and electrostatic induction mechanism to generate electricity, TENG is a promising candidate for power supply and self‐powered sensors.^[^ ^63^ ^]^ However, Using TENGs as a power source to drive functional e‐skin is difficult to collect power continuously, and the whole power‐sensing is complex, so there are few such studies.^[^ ^64^ ^]^ The basic structure of TENG is composed of triboelectric layer (tribo‐layer) and electrode. Assembled by nanocomposite membranes, Figure 2E illustrates the structure of a single‐electrode mode TENG.^[^ ^65^ ^]^ The modified PDMS film is the tribo‐layer on the top, with a strong tendency of electron acquisition. Silver nanowires (AgNWs) in the middle are evenly distributed in the thermoplastic polyurethane (TPU) nanofiber network as a stretchable electrode. At the bottom, commercial VHB taps have excellent mechanical and thermal properties and play the role of structural support and protection. For further improving the output performance of TENG, the surface of the PDMS thin film was modified by the transverse arrangement of microstructure. SEM images are illustrated in the inset of Figure 2E. Because of the increased friction area, the fabricated microstructure greatly increases the power output of TENG. The detailed working mechanism is briefly illustrated in Figure 2F, where it is combined with triboelectrification and electrostatic induction. When an active object (such as hand or foot) is in contact with a dielectric layer (PDMS film), due to the strong electronegativity of PDMS film, electrons from the active object (nylon film) are transferred to the PDMS film, resulting in an equal amount of opposite charge between the two contact surfaces. In the beginning, when the nylon membrane is separated from the PDMS membrane, a gradually increasing potential difference is generated, causing the electrons on the AgNWs electrode to flow to the ground. The electrons continue to flow until the two membranes are far apart. Then, when the two membranes approach each other, the electrons flow in the opposite direction, from the ground back to the electrode. The reciprocating cycle of contact and separation between the two triboelectric layers produces alternating current. As the contact force increases, the contact area between the two triboelectric layers will also increase, generating larger corresponding voltage output, which finally reaches 65 V saturation when the pressure is close to 6 kPa (Figure 2G). The pressure sensitivity of 9.973 mV Pa^−1^ demonstrates a well‐behaved linear response. These results indicate that the self‐powered e‐skin based on TENG has a good response to external mechanical stimulation.

### 2.2 Solar energy

Radiation from the sun on the human body has a major impact on daily life. Excessive ultraviolet radiation can lead to some diseases, such as skin cancer, pigmentation cancer, and cataracts. On the contrary, the right amount of infrared radiation can promote the local microcirculation of the human body, can strengthen the metabolism of lactic acid, increase the nutritional metabolism of muscle.^[^ ^66^ ^]^ Therefore, efficient and accurate real‐time monitoring of solar intensity has become one of the most important characteristics of e‐skin, among which, the e‐skin that can use the photovoltaic effect to self‐powered light intensity detection is more popular among people.

**FIGURE 3:**
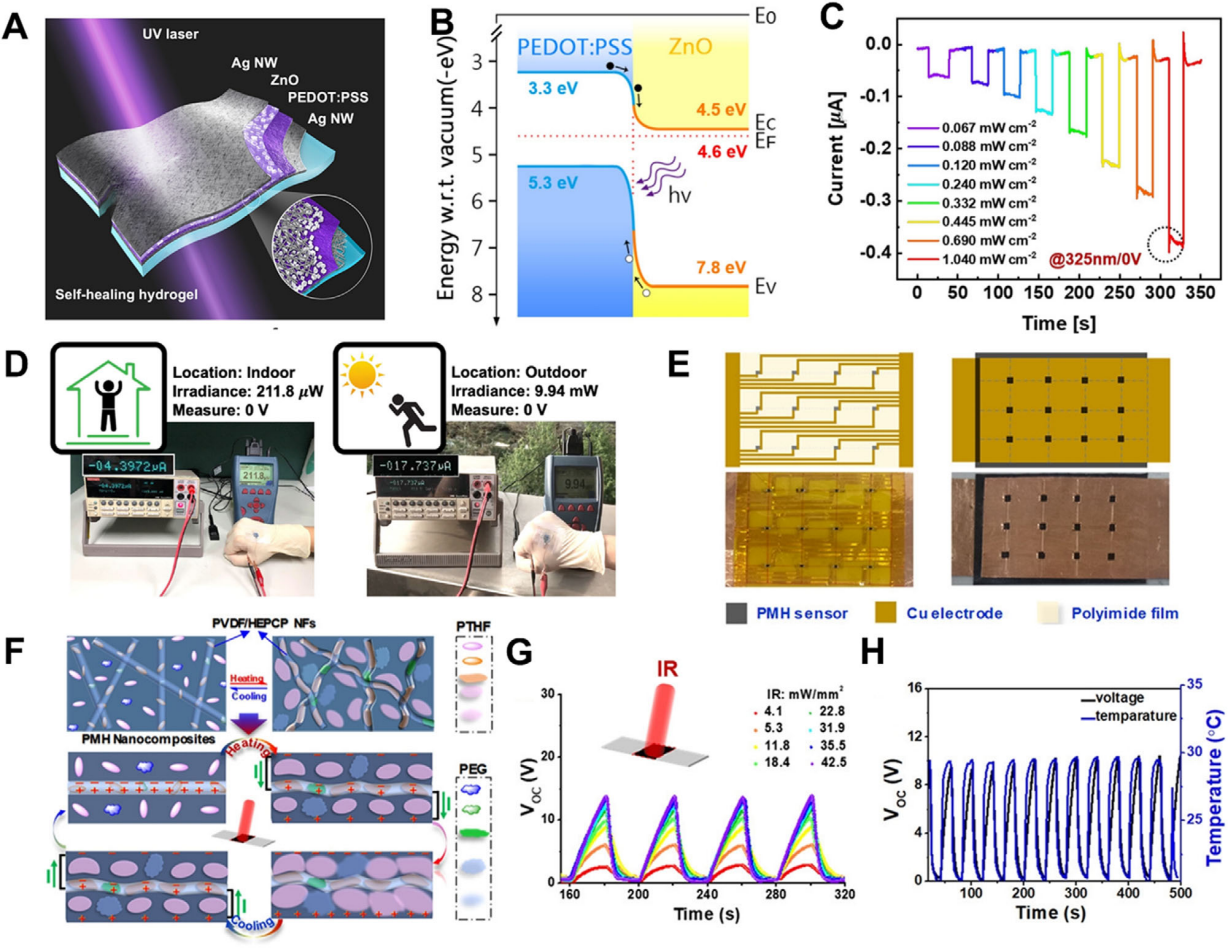
Response of electronic skin to light intensity. (A) Sketch map of the self‐powered UV photodetector. (B) Energy band chart of the PEDOT:PSS/ZnO heterojunction under light. (C) Photoresponse under varying incident light intensity values. (D) Actual testing of devices outdoors and indoors. Reproduced with permission.^[^ ^67^ ^]^ Copyright 2020, American Chemical Society. (E) Diagram and optical photographs of the infrared sensor. (F) Working mechanism of e‐skin response to IR on/off irradiation. (G) Cyclic *V*_OC_ of the photodetectors under intermittent IR lighting with different intensity values. (H) Reduplicative *V*_OC_ and temperature signals of the e‐skin under intermittent IR. Reproduced with permission.^[^ ^68^ ^]^ Copyright 2020, American Chemical Society

The photodetector is the most common self‐powered e‐skin for light intensity detection. The principle is to use the depletion layer generated by the PN junction to convert the optical signal into an electrical signal to detect the intensity of light. Tsai et al. prepared a self‐powered e‐skin for detecting ultraviolet light. The specific device schematic diagram is shown in Figure 3A.^[^ ^67^ ^]^ This device made use of ZnO and PEDOT: PSS as heterojunctions and silver nanowires (AgNWs) as conductive electrodes. The basic principle of the photovoltaic effect can be clearly understood from Figure 3B, which displays the energy band illustration of PEDOT: PSS/ZnO heterojunction under lights. When the two are in contact, due to the difference in Fermi level, electrons will flow from ZnO to PEDOT: PSS. The flow of electrons stops when the thermal equilibrium is reached, thereby forming a depletion zone at the interface. The dark current is effectively suppressed in the depletion region. As a result, it improves the detection selectivity of the device. In the light, under the action of the internal electric field of the PEDOT: PSS, the photogenerated electrons in the ZnO layer are ejected onto the AgNW electrode. At the same time, the photogenerated holes are attracted to the PEDOT: PSS, and the holes reach the electrodes. With the increase of incident light intensity, more photons will participate and generate more electron‐hole pairs, and the photocurrent response will also increase. Figure 3C represents the relationship between the output current of the self‐powered photodetector and varying light intensities. The optical response of 0.067–1.040 mW cm^−2^ under 325 nm illumination is measured, indicating that the photodetector can easily identify illumination with different light intensity and send out a reminder for human beings. To prove that this photodetector can be used in practical applications, the device was placed on a human hand and tested under mimetic indoor and outdoor UV light (Figure 3D). It can be seen that there is a big difference in the photocurrent detected under UV radiation which is very different from that detected indoors and outdoors.

Another way to measure the intensity of light is to change the crystallinity of piezoelectric material by sensing its temperature, thereby generating a voltage signal. Li et al. prepared an infrared sensor based on this principle.^[^ ^68^ ^]^ The schematic plot and optical photo of the infrared sensor can be seen in Figure 3E. Figure 3F shows the melting and recrystallization of microcrystals in the material under IR irradiation or after IR removal. Under IR light, localized and microscopic PTHF‐PEG (poly(tetrahydrofuran)‐poly(ethylene glycol)) crystal regions in the piezoelectric material melt, resulting in localized volume expansion, and then compression internal stress on PVDF nanofiber. The elastic micro‐deformation of PVDF nanofibers and the peak voltage are generated. After removing the IR light, the melted PTHF‐PEG region recrystallized, and the volume constantly shrank, thus the compressive internal stress was weakened and the opposite result was generated. As shown in Figure 3G, the PMH array produces a very obvious and rapid voltage response under infrared irradiation. Under the IR excitation of ≈ 4.1–42.5 mW/mm^2^, the open‐circuit voltage of the device increases from 2.5 to 13.3 V, so the nanocomposite is dramatically sensitive to infrared rays. From Figure 3H, we can see that real‐time cycle detection of temperature and piezoelectric voltage variations of the electronic skin sensor system is realized by intermittent IR lighting. Two kinds of response are perfectly matched, that is, the infrared detecting mechanism of the device is connected with its own temperature change.

### 2.3 Electromagnetic energy

Radio frequency identification (RFID) tags also have a significant place in the self‐powered sensor field. Unlike normal active RFID tags, which need to be connected to an external power source (such as a battery) to power the radio frequency,^[^ ^69^, ^70^ ^]^ passive RFID tags can power themselves by extracting energy from the active radio frequency source and using the inductance of the ring‐shaped antenna to power for itself.^[^ ^71^, ^72^ ^]^ This allows the label to be made into different shapes such as sheets or hooks to be applied in different environments, and the device is used almost forever. RFID tags are a very basic radio frequency energy harvesting solution available on the market. Because the passive RFID tag is thin and flexible, it can be easily attached anywhere, such as embedded in the human body for health monitoring or attached to the wall for gas monitoring.^[^ ^73^ ^]^

**FIGURE 4:**
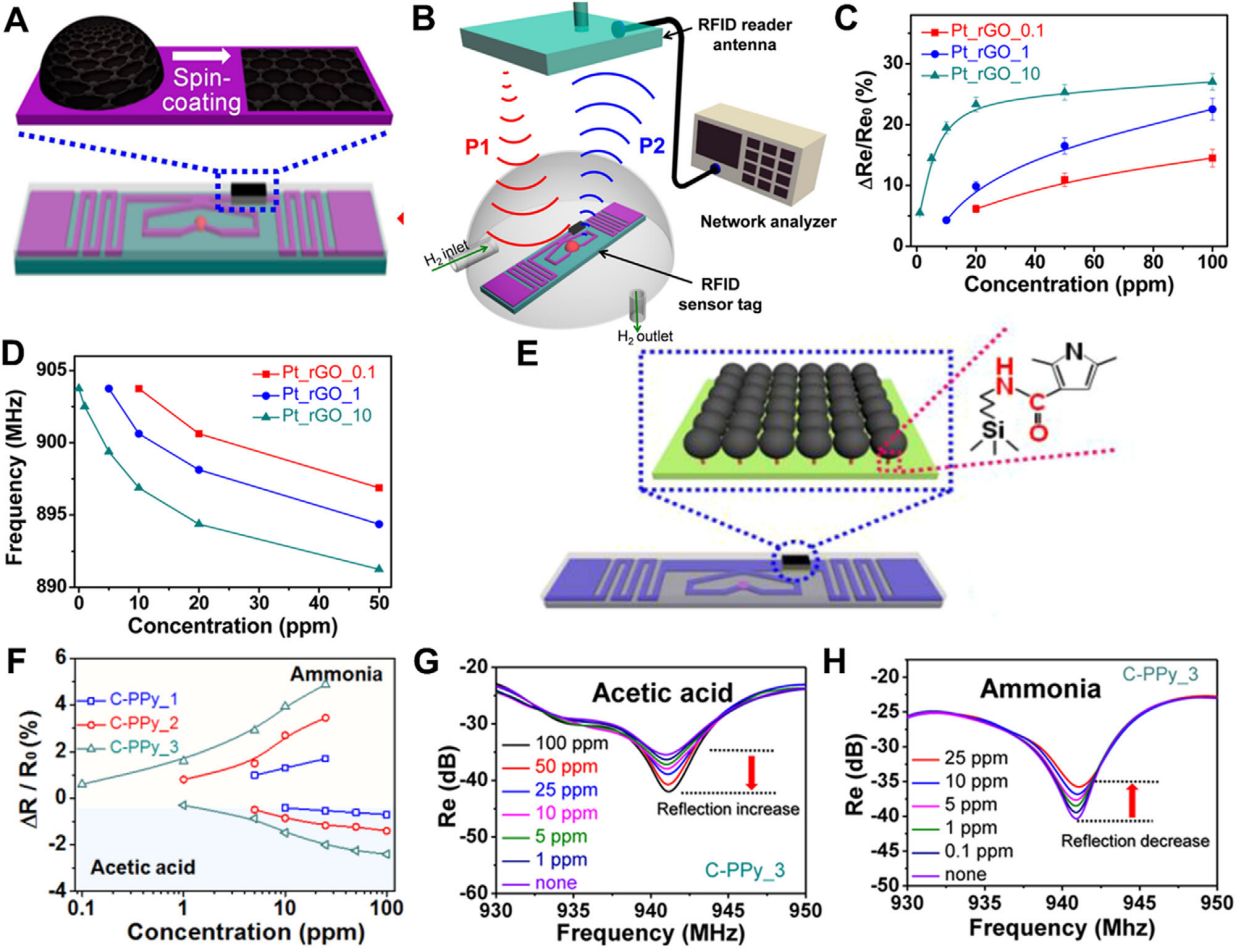
Response of electronic skin to light intensity. (A) Schematic diagram of the RFID tag. (B) Application of the RFID‐based wireless sensor system. (C) Reflection calibration and (D) Phase shift calibration curves of the three tags against H_2_ concentration. Reproduced with permission.^[^ ^74^ ^]^ Copyright 2015, American Chemical Society. (E) Chart of the RFID sensor tag with carboxyl functional groups covalently bonded to the aluminum tag. (F) Relationship between NH_3_ concentration and reflectance change. Change in reflectance properties of wireless sensors response to (G) acetic acid and (H) ammonia. Reproduced with permission.^[^ ^75^ ^]^ Copyright 2016, American Chemical Society

In order to achieve hydrogen detection at room temperature, a wireless smart sensor based on RFID has been reported. This sensor consists of a network analyzer‐connected RFID antenna as a query reader, and reduced graphene oxide (rGO) decorated by platinum (Pt) as a sensing label (Figure 4A).^[^ ^74^ ^]^ In the process of hydrogen detection, a robust affinity between the modified Pt and hydrogen is generated, which changes the resistivity of the antenna and the reflectivity of the RFID tag. When the RFID sensor tag is located in the electromagnetic field of the RFID antenna, the network analyzer emits an inquiry signal P_1_, which is feedbacked to the RFID reader antenna as P_2_. The target monitor can thus be analyzed based on the reflected signal (Figure 4B). Figure 4C illustrates the sensor reflection calibration (normalized reflection change) against different H_2_ concentrations. For all sensor label samples, the normalized reflection of radio waves adds when H_2_ concentration adds. However, when the concentration exceeds 50 ppm, the amount of change in reflection decreases, indicating that the sensor label tends to saturate with H_2_. Besides, the corrected reflection phase shows the same tendency, too (Figure 4D).

Carboxyl group functionalized polypyrrole (C‐PPy) nanoparticle is also a compound that is very sensitive to gases. Jun et al. prepared an RFID‐based wireless sensor system with this compound. This system consists of an RFID reader antenna and a commercial C‐PPy nanoparticles‐coated RFID tag (Figure 4E).^[^ ^75^ ^]^ In Figure 4F, it can be found that the C‐PPy sensor is very sensitive to both NH_3_ and acetic acid, and is positively correlated with the concentration of NH_3_, while the concentration of acetic acid is opposite. During the sensing process, the network analyzer‐connected RFID reader antenna emits a signal and activates the sensor, then the emitted signal is fed back into the reader antenna, and the network analyzer monitors the reflected signal in real‐time. In an ammonia atmosphere, the resistance change in the chemical response material results in an impedance mismatch between the dipole tag antenna and the IC (integrated circuit) chip. Consequently, the backscattered signal changes can be detected by the network analyzer. Figures 4G and 4H show the changes in the backscattering power level of the RFID sensor tag in response to acetic acid and ammonia, respectively, indicating that the RFID sensor shows ultra‐high sensitivity to ammonia and acetic acid, with detection concentrations as low as 0.1 and 1 ppm, respectively. Because the sensor is passive, the fabrication of the device does not require any shape, so this RFID‐based sensor is very suitable for e‐skin of gas sensors.

### 2.4 Thermal energy

Temperature is one of the most exposed physical quantities in our daily life. People's daily life, the survival, and reproduction of animals and plants are closely related to the temperature of the surrounding environment. Petroleum, aerospace, archival preservation, and other fields also have high requirements on temperature. Due to the universality of temperature measurement, temperature sensors occupy a very large proportion of various sensors. In the last decade, with the introduction of the thermoelectric effect, self‐powered temperature sensors have also arisen. Based on the Seebeck effect, the thermoelectric effect can convert heat into electricity, which creates a *V*_oc_ between thermocouples (a combination of two different thermoelectric materials).^[^ ^76^ ^]^ The heat generated by the temperature gradient of different heat sources, for example, the body or environment, can be utilized by thermoelectric materials, which is useful for health monitoring.^[^ ^77^ ^]^

**FIGURE 5:**
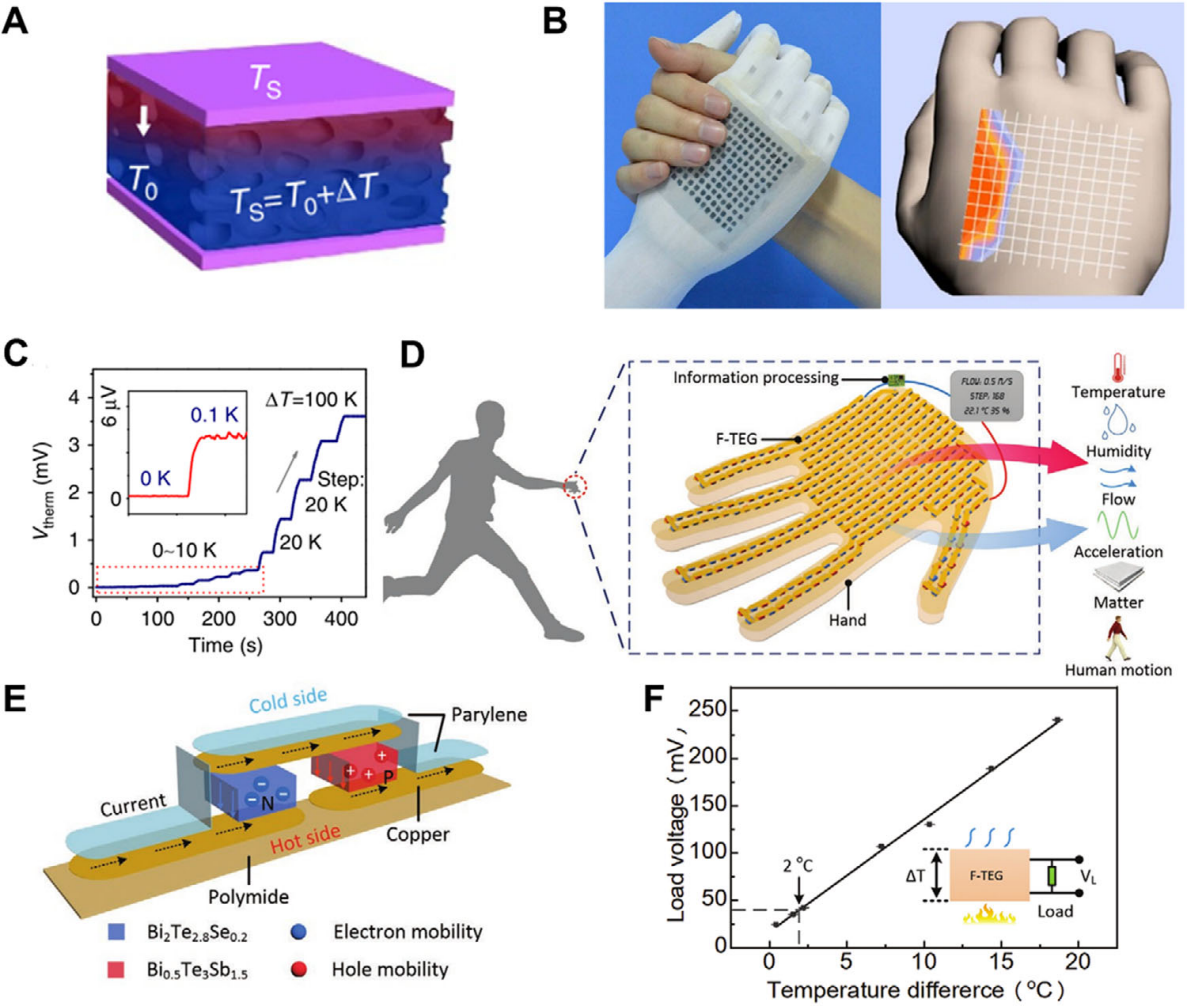
Response of electronic skin to temperature. (A) Schematic chart of temperature sensing mechanism. (B) Optical figure of an arm‐wrestling between a prosthetic hand and an adult woman. The right figure shows the temperature mapping plots of pixel signals during the arm‐wrestling. (C) Output voltage values of the sensor to a biased temperature gradient range from 0 to 100 K. Reproduced with permission.^[^ ^78^ ^]^ Copyright 2015, Springer Nature. (D) Schematic of the hand‐shaped e‐skin sensing system. (E) An amplified view of a thermoelectric unit. (F) The relationship between load voltages and different temperature gradients. Reproduced with permission.^[^ ^79^ ^]^ Copyright 2020, Wiley‐VCH

Although the PEDOT: PSS coating is not as efficient as ordinary inorganic thermoelectric materials, its flexibility makes it ideal for self‐powered e‐skin. When the device is close to an object, the temperature difference between the object and device can be detected through the typical thermoelectric mechanism, resulting in an instantly measuring of the surface temperature of objects just like human skin (Figure 5A).^[^ ^78^ ^]^ On this basis, a simple stamp‐printing approach was utilized to construct a flexible temperature sensor array in translucent fabric gloves (Figure 5B). Due to its excellent flexibility and biocompatibility, the array of temperature sensors can be worn on a prosthetic hand. When an adult female wrestles with the prosthetic hand, the device can successfully detect and collect spatial resolution images with temperature imaging characteristics. At the same time, the prepared device is also capable of very small temperature differences. Figure 5C shows the relationship between the measured voltages and temperature gradients (0.1 to 100 K). Even the temperature difference of 0.1 K can be easily measured by the device, indicating the very accurate temperature resolution of the temperature sensor.

Yuan et al. made a human hand‐shaped e‐skin and attached it to a hand.^[^ ^79^ ^]^ A skin‐like thermoelectric generator (F‐TEG), as the core component, is worn on the hand and can not only sense temperature but also obtain energy from body heat as an energy source for multi‐sensory receptors (Figure 5D). The sensing principle of F‐TEG is shown in Figure 5E. F‐TEG consists of p‐type (Bi_0.5_Sb_1.5_Te_3_) and N‐type (Bi_2_Te_2.8_Se_0.2_) thermoelectric grains with high thermoelectric conversion efficiency assembled on a flexible polyimide (PI) substrate. The hot and cold sides of the device are linked in series and parallel through the Cu electrode. When there is a temperature difference, the electrons at the hot side move violently and diffuse to the cold side, thus causing the thermoelectromotive force. When different temperature differences are applied on the hot and cold sides, the e‐skin can achieve a temperature sensing sensitivity of 150 mV °C^−1^ after amplifying signals (Figure 5F). The superior sensing performance of this sensor, as well as its strengths of low cost and mass production, make it suitable for use in health monitoring components.

### 2.5 Chemical energy

In addition to the temperature of the environment and the mechanical energy generated by the human body, e‐skin can also be a source of chemical potential energy. Biofuel cells (BFCs) can extract chemical potential energy generated by human body fluids, such as saliva, urine, sweat, and blood.^[^ ^80^, ^81^ ^]^ In simple terms, based on biocatalytic oxidoreductase reactions, BFCs transform chemical energy into electrical energy. By observing the amount of electricity generated, we can accurately understand the content of biological components in the human body, and then make timely feedback to human health.

**FIGURE 6:**
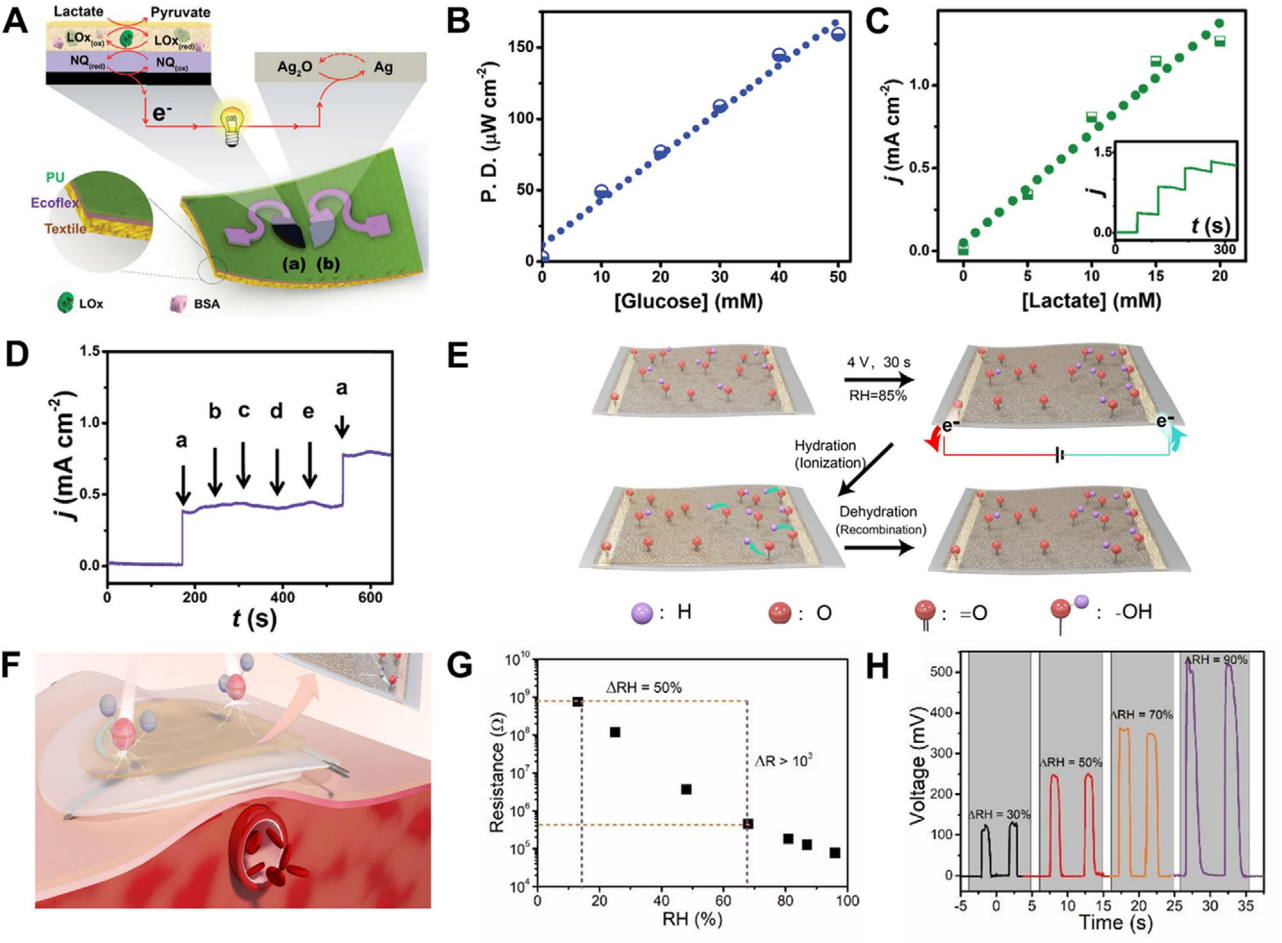
Response of electronic skin to chemical energy. (A) The compositions of the stretchable lactate BFC. (B) Power‐concentration calibration chart of glucose. (C) The self‐generated current response against lactate concentration. (D) Response of the BFC sensor to (a) 5 mmol L^−1^ lactate, (b) 84 mmol L^−1^ creatinine, (c) 10 mmol L^−1^ ascorbic acid, (d) 0.17 mmol L^−1^ glucose, and (e) 59 mmol L^−1^ uric acid. Reproduced with permission.^[^ ^84^ ^]^ Copyright 2016, The Royal Society of Chemistry. (E) Polarization of PDA with applied voltage in a humid environment. (F) The self‐powered wearable sensing system. (G) Resistance of PDA membrane against RH variation. (H) Output voltage of the device to four different RH. Reproduced with permission.^[^ ^85^ ^]^ Copyright 2019, American Chemical Society

Biofuel cells are classified according to their biochemical reactions and the properties of their electrodes. Enzymatic BFCs uses enzymes to catalyze the oxidation of fuel, but the life of the enzymes is very short and can only partially oxidize fuel.^[^ ^82^ ^]^ Microbial BFCs use living cells to catalyze fuel, but fuel needs to be transported through cell membranes, so the efficiency is very low. As a result, microbial BFCs are limited by low power density.^[^ ^83^ ^]^ Therefore, using BFC as a sensor for detecting chemical substances is a very good choice. Through screen printing, flexible textile BFCs are fabricated with the ability to detect biofuels (Figure 6A).^[^ ^84^ ^]^ The bioanode is used as a redox mediator by a single enzyme, that is, glucose oxidase (GO*_X_*) or lactate oxidase (LO*_x_*), and 1,4‐naphthoquinone (NQ). Once the biofuel (such as glucose or lactic acid) is added, the biofuel is oxidized at the anode by the enzyme, and electrons are liberated. In the cathode chamber, the Ag_2_O receives these electrons to fulfill the power circuit. The power density of a biofuel cell increases with the increasing concentration of added biofuel (glucose). The attractive conductivity of the carbon nanotubes in the special inks achieves high power density, which facilitates the electrons flowing between the anode and cathode. The power density linearly adds with glucose concentrations of 0 to 50 mM with sensitivity of 3.14 μW cm^−2^ mM^−1^, as shown in Figure 6B. By replacing the anode material with LO*_X_*, the biofuel cell can respond to lactate concentration in a timely manner (Figure 6C). At the same time, even in the case of common coexistence interference, fuel cell still has high selectivity. When the self‐powered sensor is in a different environment, it only responds to lactic acid (Figure 6D). Therefore, the system can monitor analytes (fuels) in real complicated matrices without any disturbance.

There is also extensive chemical potential energy in the processes of spontaneous diffusion (such as water and ion diffusion), which is a very viable way to power self‐powered sensors. Obtain g‐PDA to construct hydroxyl with gradient distribution by applying an opposite voltage between electrodes, as shown in Figure 6E.^[^ ^85^ ^]^ Theoretically speaking, the oxygen‐containing groups with gradient distribution will be ionized, and the gradient‐free cation (H^+^) and locally confined anion for hydration will be released. Therefore, the gradient distribution of ion concentration drives the migration of free cation (H^+^), endows the g‐PDA film with self‐charging capability (Figure 6F). For quantitatively assessing the ability of the PDA membrane to capture water, real‐time resistance monitoring was carried out. As the relative humidity (RH) was added (Figure 6G), the ionic conductivity of PDA increased significantly. When no additional voltage is applied to test the device's induction to humidity, it can be seen from Figure 6H that a series of open‐circuit voltages caused by moisture is gradually increased as RH changes from 5% to 35%. The device's unique treatment of the functional layer makes the self‐powered humidity e‐skin the first to be proposed, providing a new way for self‐powered multifunctional integrated e‐skin.

## 3 INTEGRATED ENERGY

The self‐powered multifunctional e‐skin involved collects energy from mechanical motion, light, and even chemical reactions without external energy to generate sensing signals. Under the circumstances, it acts as both an energy harvesting device and a sensor. It's worth emphasizing that the difference between self‐powered e‐skin here and the self‐powered e‐skin system. The former could generate sensing signals autonomously without consuming external energy, but still requires an external power source to capture and transmit sensing signals. The latter realizes the autonomous driving of the whole system by integrating energy harvesting, self‐ sensing, signal processing, and transmission modules efficiently.

**FIGURE 7:**
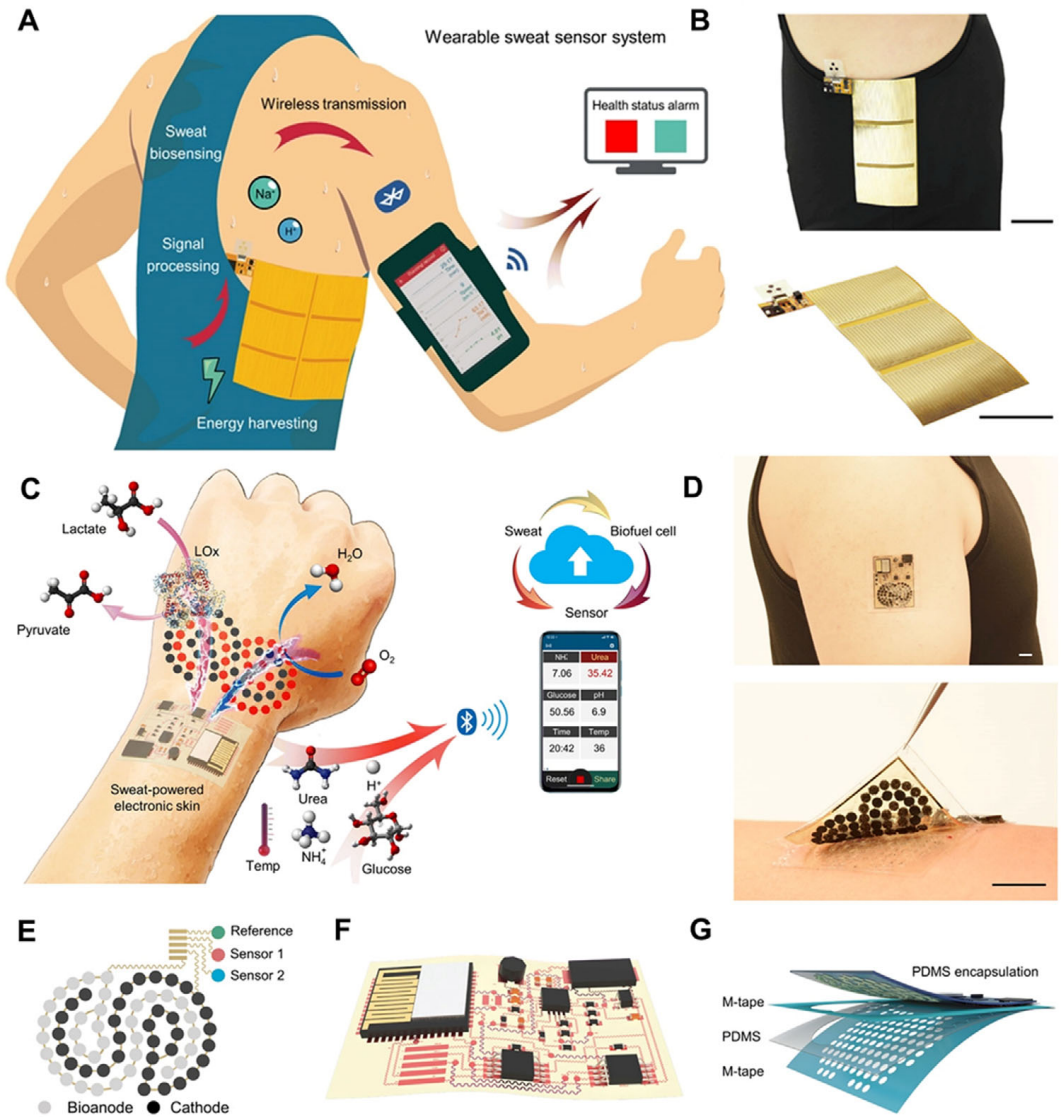
Integrated self‐powered electronic skin. (A) Diagram of the sensor system for real‐time health monitoring. Reproduced with permission.^[^ ^86^ ^]^ Copyright 2020, American Association for the Advancement of Science. (B) Optical photos of the sweat sensor platform attached on a human torso. (C) Diagram of the biofuel‐powered e‐skin. (D) Optical photographs of the e‐skin on a healthy adult's arm. Schematic plots of (E) the flexible sensor and (F) the soft e‐skin interface. (G) System‐level encapsulation for biofluid sampling. Reproduced with permission.^[^ ^87^ ^]^ Copyright 2020, American Association for the Advancement of Science

Among many physiological signals, sweat containing complex physiological information has become a potential analysis target for noninvasive continuous sensing of the human body. By collecting the mechanical energy in the process of motion and using it as a sweat sensor platform, they provide a new research idea for fully integrated self‐powered e‐skin. As shown in Figure 7A, Song et al. use all‐in‐one flexible circuit board processing technology to achieve the integrated preparation of freestyle TENGs and flexible circuit modules, the platform can efficiently capture the mechanical energy in human motion and convert it into electrical energy, drive the stable work of electrochemical sensing units, and realize wireless signal transmission and dynamic indicator monitoring of biomarkers (e.g., pH, sodium ions) in sweat.^[^ ^86^ ^]^

However, using a freestyle TENG (Figure 7B) to collect energy makes the platform bulky, less flexible, and comfortable. On this basis, they also used biomass energy in sweat as energy, using biofuel cells as a power supply device and integrating biological and physical sensors on ultra‐thin and transparent polyimide (PI) substrates to build a new generation of self‐powered e‐skin. Key metabolic biomarkers such as glucose, urea, NH_4_, and pH are collected and personalized information is wirelessly transmitted to the user interface through Bluetooth Low Energy. As shown in Figure 7C, the biofuel cell combines integrates zero‐dimensional to three‐dimensional nanomaterials and modifies the corresponding lactic acid oxidase and platinum cobalt alloy nanoparticles to obtain 3.5 mW cm^−2^ power density directly from sweat and to provide continuous energy to the e‐skin for 60 h.^[^ ^87^ ^]^

Based on the abundant energy supply, the researchers further optimized the efficiency of energy utilization, controlling the Bluetooth module to complete wireless data transmission by switching between sleep/activation modes to reduce the energy demand of the e‐skin and extend continuous working hours. As shown in Figure 7D, the device is so thin that it does not even feel it when worn. It is composed of two main components: (i) a flexible electrochemical patch containing biosensors (Figure 7E) for energy collection and detection in body sweat. (ii) Ultra‐thin polyimide plates containing rigid electronics (Figure 7F) to realize power management, signal handling, and wireless communication. Electronic devices are packaged in polydimethylsiloxane (PDMS) (Figure 7G) to avoid sweat contact with electronic devices. Our sweat contains concentrated lactic acid, which is absorbed by the fuel cell of the e‐skin. The e‐skin is powered by a biofuel cell, which produces sufficient as well as sustained power.

## 4 COUPLING OF SELF‐POWERED EFFECT IN ELECTRONIC SKIN

Commendable advances in the progress of e‐skin have been realized recently. An e‐skin that can recognize a single physical parameter has been realized. However, the e‐skin needs to truly realize the integration of human skin's sensing of physical parameters including touch, temperature, humidity, etc. Only in this way can it be truly used in fields of human–machine interface, medical and health, and so on. Single‐effect self‐powered e‐skins are apparently unable to meet application requirements. There is an imperative need to develop coupled, multifunctional, integrated, and sustainable e‐skin.^[^ ^88^ ^]^ Combining and integrating the above‐mentioned multiple energy effects is an effective way to develop a coupled self‐powered multifunctional e‐skin. For the multifunctional coupling e‐skins, the problems that need to be solved are as follows.

1. Integrate different energy effects into one structure, while ensuring the flexibility, lightweight, and comfort of the device;
2. The e‐skin system is capable of sensing multiple signals at the same time, without signal interference. Until now, plenty of studies have conducted systematic research on the material preparation, device assembly, and output signal analysis of the coupled self‐powered e‐skin.

In this section, according to the number of coupling effects, we divide the recent progress into two parts, “double coupling effects” and “multiple coupling effects,” for facilitating the reader's reading.

### 4.1 Double coupling effects

#### Tribo‐piezoelectric effects for pressure and tactile sensing

**FIGURE 8:**
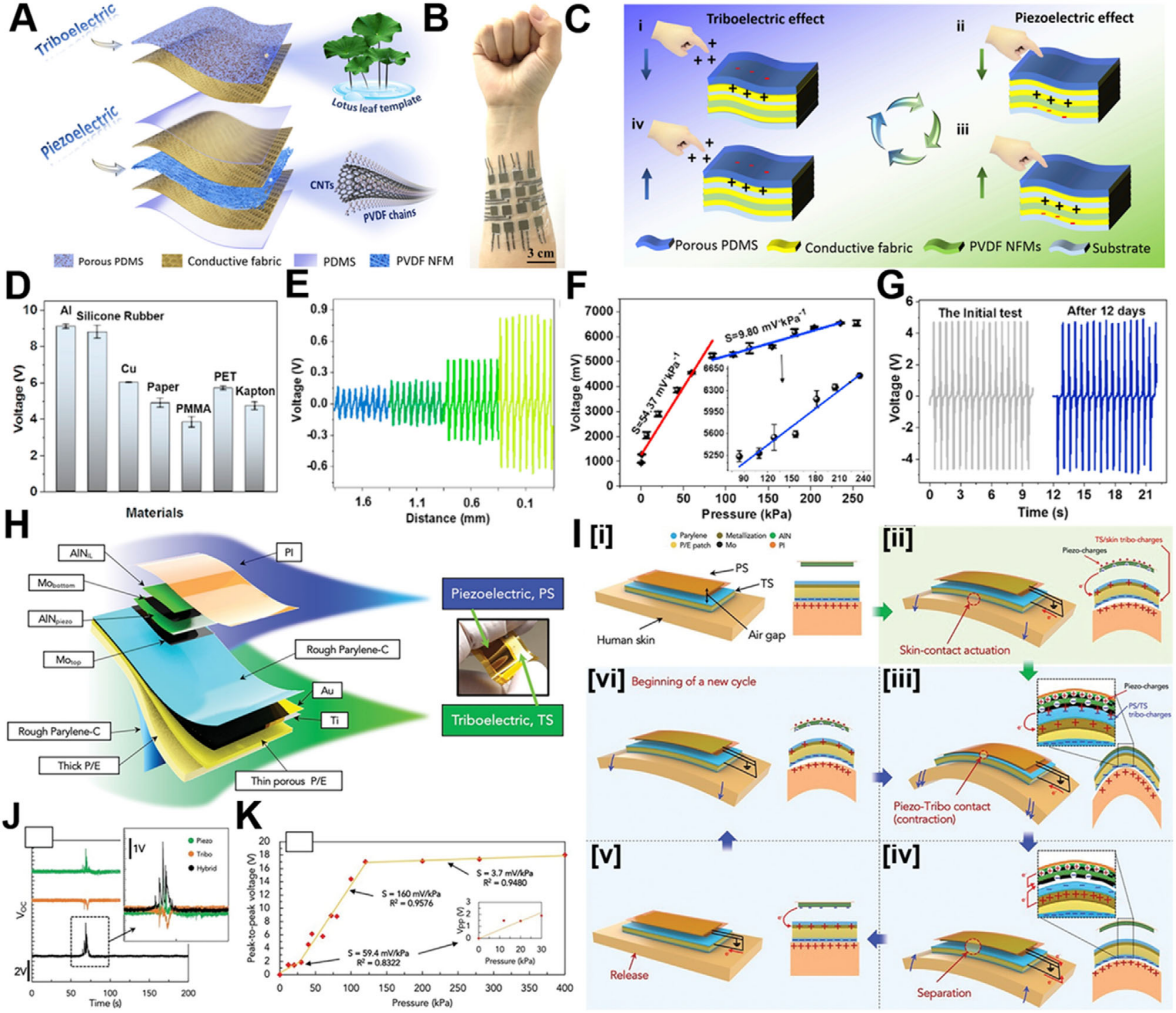
Piezo‐triboelectric coupling affects self‐powered electronic skin. (A) Structural sketch, (B) optical photograph, (C) working principle of the hybrid e‐skin. (D) Sensing performances of different materials. (E) Voltage signal of the e‐skin with different distances between PI. (F) Pressure sensitivity of the 1 cm × 1 cm e‐skin. (G) The stability of the hybrid sensor. Reproduced with permission.^[^ ^93^ ^]^ Copyright 2020, Elsevier Ltd. (H) Exploded view of the hybrid sensor. The inset is an optical picture of the sensor. (I) Working principle of the e‐skin with the contraction‐release cycle. (J) Piezoelectric and triboelectric *V*_oc_ signals, and the overlapped hybrid signal. (K) Voltage output of the wearable hybrid sensor under pressure on skin. The corresponding sensitivities are marked in the chart. Reproduced with permission.^[^ ^94^ ^]^ Copyright 2021, Wiley‐VCH

Both triboelectric effect and piezoelectric effect can convert mechanical energy into electrical energy.^[^ ^89^, ^90^, ^91^, ^92^ ^]^ Through combining them, the advantages of the two effects can be utilized simultaneously to broaden the scope of application. Zhu et al. developed a self‐powered e‐skin combining triboelectric effect and piezoelectric effect and achieved a wearable multifunctional sensor.^[^ ^93^ ^]^ Figure 8A is the structure design diagram of the hybrid e‐skin. The triboelectric material is a rough and porous polydimethylsiloxane (PDMS) film designed with a natural lotus leaf as the template. Multi‐walled carbon nanotubes (MWCNTs) are doped into PVDF nanofibrous film, which is acted as the piezoelectric material, and the electrodes are flexible conductive fabrics. Figure 8B is the optical picture of the prepared sensor array. Figure 8C depicts the mechanism of the tribo‐piezoelectric coupling effects. Through the triboelectric effect, the e‐skin can distinguish between different contact materials (Figure 8D) and perform non‐contact distance recognition (Figure 8E). Figure 8F displays that under the synergistic effect of the triboelectric and piezoelectric effect, the device displays high pressure sensitivity of 54.37 mV kPa^−1^ and 9.80 mV kPa^−1^ in 0–80 kPa and 80–240 kPa, respectively, and the device demonstrates excellent stability (Figure 8G). In addition, Mariello et al. also combined the two generator mechanisms to develop a conformal tribo‐piezoelectric e‐skin with biocompatible materials.^[^ ^94^ ^]^ Figure 8H,I are the schematic diagrams of the structure and mechanism of the device, respectively. Its working mechanism is the three effects of piezoelectric, skin‐contact‐actuation, and piezo‐tribo hybrid contact. As shown in Figure 8J, the triboelectric signal and the piezoelectric signal overlap into one pressure signal, yielding a hybrid output. The device has the characteristics of high sensitivity and a wide detection range, and its highest pressure sensing sensitivity is 160 mV kPa^−1^ (Figure 8K) between 50 and 120 kPa. The e‐skin has a variety of functions, which can recognize human gait walking, identify hand gestures through a sensor array system, and monitor joint movements of the human body (neck, wrist, elbow, knee, ankle, etc.).

#### Temperature and pressure dual‐parameter detection

**FIGURE 9:**
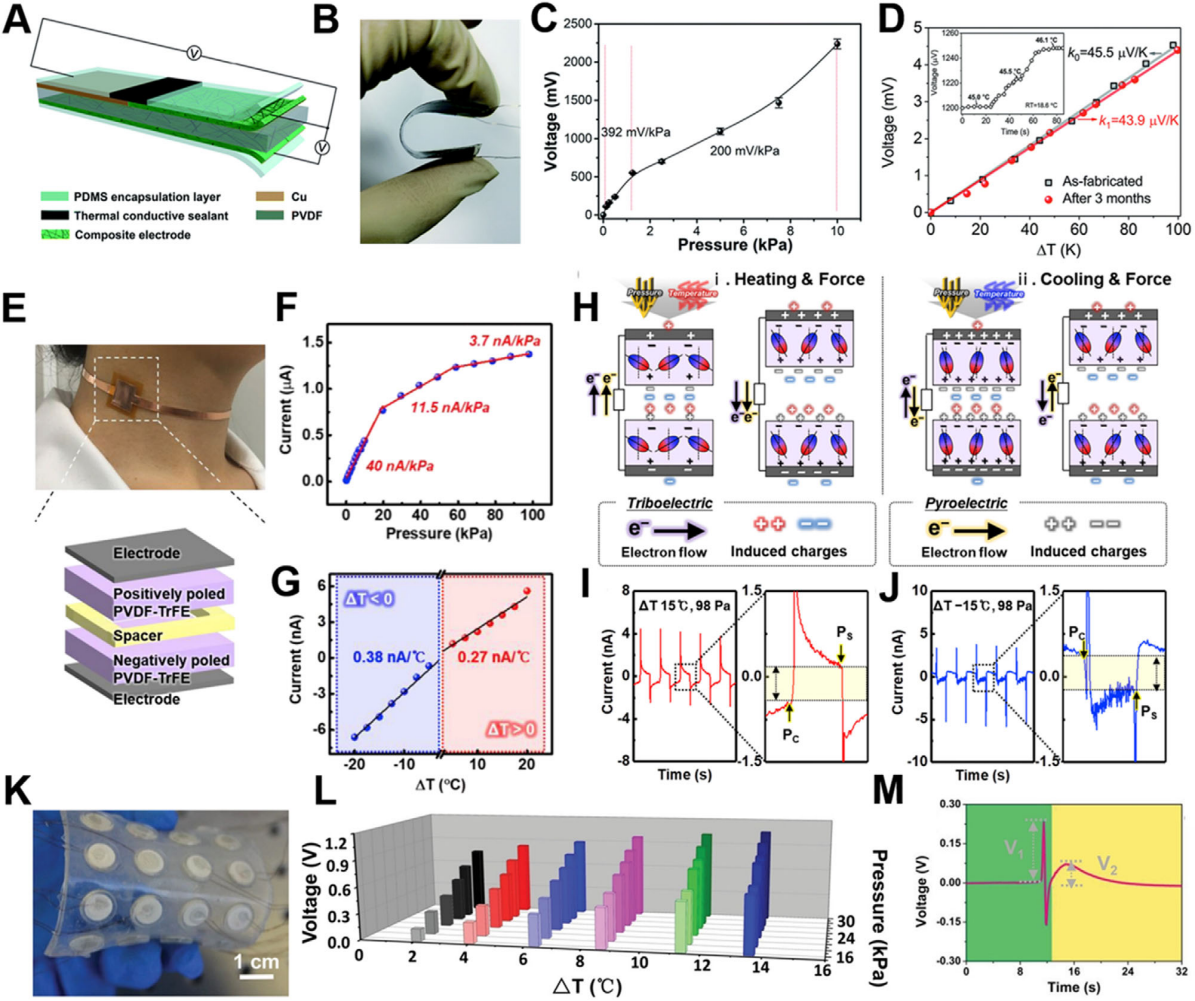
Double coupling affects electronic skin for simultaneous temperature and pressure sensing. (A) Structure of the temperature‐pressure dual‐parameter sensor and the sensing mechanism. (B) Optical photo of the folded sensor. (C) Sensitivity of the sensor under varying applied pressure. (D) The relationship between the sensor against varying temperature differences detected. The inset shows the temperature sensitivity. Reproduced with permission.^[^ ^95^ ^]^ Copyright 2019, The Royal Society of Chemistry. (E) Photographic and structural graphs of the inversely polarized P(VDF‐TrFE) based sensor. (F) Pressure and (G) temperature response of the device. (H) Diagram of triboelectric and pyroelectric mechanism. Current signals in (I) heating and (J) cooling states. Reproduced with permission.^[^ ^97^ ^]^ Copyright 2020, Elsevier Ltd. (K) Photograph of bendable Ag/BTO/Ag pyro‐piezo‐electric sensing array. (L) Histogram of voltage outputs against varying temperature gradients and different applied pressures. (M) Voltage curves when the finger touches sensor array. Reproduced with permission.^[^ ^98^ ^]^ Copyright 2019, Wiley‐VCH

Temperature and pressure are the most basic physical perception parameters of the multifunctional electronic skin. Many studies have shown that the e‐skin can simultaneously monitor temperature and pressure through the coupling of energy effects. As Figure 9A,B demonstrates, Zhu et al. proposed a flexible active dual‐parameter sensor with a sandwiched structure, which simultaneously detects temperature and tactile stimulation through piezoelectric and pyroelectric effects without signal interference.^[^ ^95^ ^]^ The piezoelectric material used is PVDF. The ternary polyaniline (PANI)‐based composite with tellurium nanorods and MWCNTs encapsulated as fillers are used as the thermoelectric material as well as the electrode. Figure 9C illustrates that the e‐skin has high sensitivity (392 and 200 mV kPa^−1^) in the low‐pressure region of 0–10 kPa. In the meantime, it also has a high temperature sensitivity (45.5 μV K^−1^) with fast response time (1.2 s) (Figure 9D). The device has good stability over months and the pyroelectric signal and the piezoelectric signal are measured separately, so there is no signal interference at all. This group later used a 3D processing technique to further optimize the structure of the device. They constructed a vertically architectured e‐skin, of which the temperature sensing sensitivity is increased to 109.4 μV K^−1^, and the response time is shortened to 0.37 s.^[^ ^96^ ^]^ Besides, Temperature and pressure can also be measured by other energy effects. For example, Shin et al. used the pyroelectric and triboelectric effects to simultaneously sense temperature and pressure.^[^ ^97^ ^]^ In Figure 9E, PVDF‐TrFE with the opposite polarity is used as the triboelectric materials, combined with their pyroelectric characteristics, can realize the highest pressure sensitivity of 40 nA kPa^−1^ in the wide pressure measurement range and temperature sensitivity of 0.38 and 0.27 nA °C^−1^ in cooling and heating states, respectively (Figure 9F,G). Using the response time difference between the pyroelectric and triboelectric signal, the two signals can be recorded separately, realizing simultaneous dual‐function sensing of the e‐skin (Figure 9H–J). According to the principle of the response time difference, the pyroelectric effect can also be combined with the piezoelectric effect to realize real‐time monitoring of pressure and temperature. Song et al. used the ferroelectric material BaTiO_3_ (BTO) with both energy effects to achieve dual sensing of temperature and pressure (Figure 9 K–M).^[^ ^98^ ^]^ The device is Ag/BTO/Ag hierarchical structure, and the sensitivity to temperature and pressure are 0.048 V °C^−1^ and 0.044 V kPa^−1^, respectively. This sensor array can be used for real‐time intelligent recognition of finger touch sensing.

**FIGURE 10:**
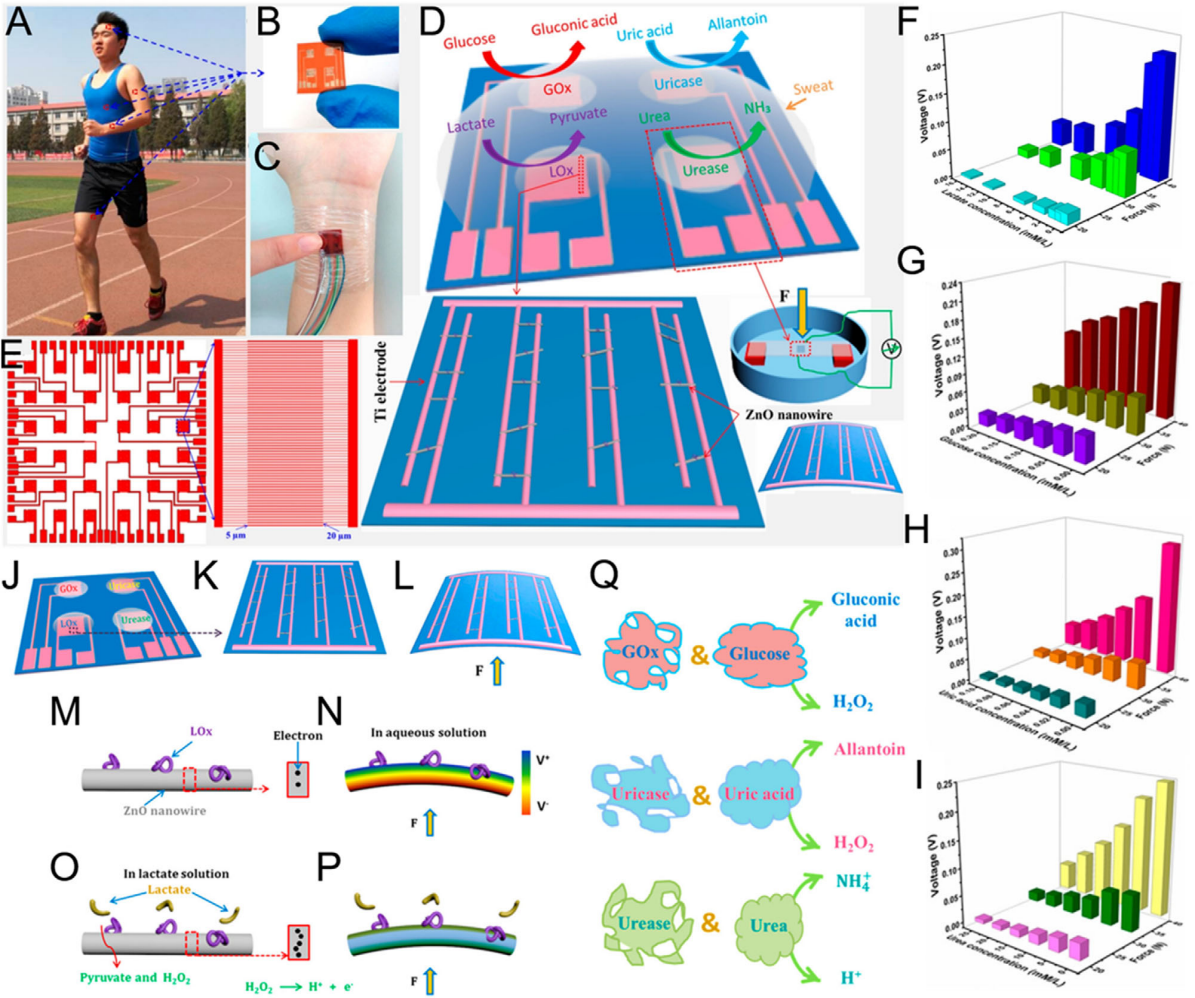
Piezo‐biosensing coupled self‐powered e‐skin. (A) The e‐skin worn on human body for detecting perspiration ingredients. (B,C) Phtographs of the e‐skin attached on wrist). (D) Diagram of measuring the concentration of lactate, glucose, uric acid, and urea. (E) Overall picture of the e‐skin. (F–I) Relationships between the output voltages of different sensing units (modified by LO*_x_*, GO*_x_*, uricase, and urease, respectively) and four sweat components ((F) lactate, (G) glucose, (H) uric acid, (I) urea) concentration under various forces (20, 32, 40 N). (J–L) Sensing units with four enzymes modification on the interdigital electrodes can be bent with stress. LO*_x_*/ZnO nanowire in (M) pure water and (O) lactate aqueous solution without stress. Piezoelectric signal in (N) pure water and (P) lactate aqueous solution generated by stress. (Q) Enzymatic reactions between GO*_x_* and glucose, uricase and uric acid, urease, and urea, respectively. Reproduced with permission.^[^ ^99^ ^]^ Copyright 2017, American Chemical Society

Except for the above coupling situations, self‐powered e‐skin can also combine the power generation effect with chemical reactions to construct a self‐powered analysis system. Han et al. of Northeastern University combined the piezoelectric effect with the enzyme reaction to achieve a self‐powered noninvasive e‐skin to realize sweat measurement with enzyme/ZnO nanoarrays.^[^ ^99^ ^]^ The piezoelectric pulse of the ZnO nanowire sensing unit is used as the pressure sensing signal and the power supply for the entire sensing system. Four different sensing areas are constructed using four different enzymes, which are used to detect the concentrations of lactate, glucose, uric acid, and urea (Figure 10A–E). It can be obtained from Figure 10F–I that the outputs of the sensing units can change with the applied forces and concentrations of the four components in sweat, without interference between variables. The piezoelectric‐enzymatic‐reaction coupling of enzyme/ZnO nanowires is the working principle of the system (Figure 10J–Q).

### 4.2 Multiple coupling effects

**FIGURE 11:**
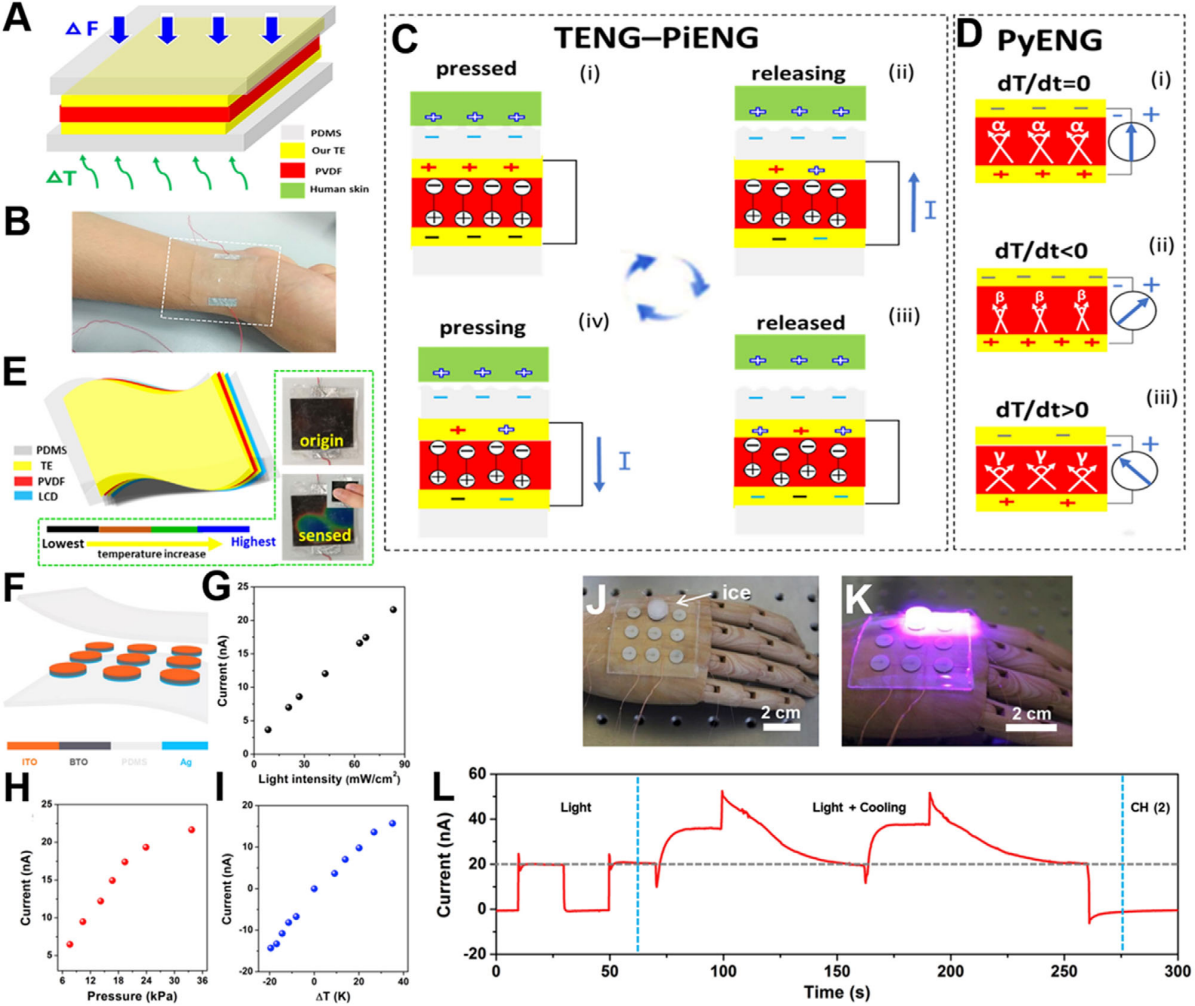
Multiple coupling effects self‐powered e‐skin. (A) Schematic and (B) picture of the coupled device. (C,D) Working principles of TENG‐PiENG (piezoelectric nanogenerator), and the PyENG (pyroelectric nanogenerator). (E) The smart visualized thermometer consists of the nanogenerator and LCD. Reproduced with permission.^[^ ^100^ ^]^ Copyright 2018, Elsevier Ltd. (F) Structure of the sensor system. Relationships between output current of the e‐skin and (G) various light (H) intensities, pressures, and (I) temperature gradients. Optical photograph of (J) ice placed on the e‐skin, (K) the e‐skin under light of 405 nm, and ice cooling simultaneously. (L) Current values of the e‐skin under light of 405 nm only and simultaneous light and cooling. Reproduced with permission.^[^ ^101^ ^]^ Copyright 2020, Elsevier Ltd

Compared with the double coupling effects, the multiple coupling effects e‐skin faces more challenges. Creating an e‐skin with human‐like multifunctional abilities is an inevitable trend for future wearable electronics. Sun et al. integrated triboelectric, piezoelectric, and pyroelectric effect into one structure, and proposed a novel, flexible, biocompatible hybrid nanogenerator (HNG) with three effects coupled.^[^ ^100^ ^]^ This work arranges silver nanowires into a leaf venation‐like network. The as‐prepared low‐resistance transparent electrode provides the device with high and adjustable transparency. PVDF with piezoelectric and pyroelectric features and micro‐structured PDMS with protection and friction properties are utilized to make the HNG (Figure 11A,B). Figure 11C,D is the coupling mechanism of the three effects. According to Figure 11E, by combining this HNG with thermochromic thin liquid crystal (LCD) film which changes its colors with different temperatures, a visualized thermometer can be realized and used for medical diagnostics. The transparent HNG can be conformally worn on body and track multiple physiological signs, such as breath, heartbeat, swallow, and pulse in real‐time. In addition, integrating three different energy effects into a same device can reach the monitoring of three physical signs. Zhao et al. coupled photovoltaic, piezoelectric, and pyroelectric effects into a one‐structure‐based nanogenerator for simultaneously acquiring 405 nm light, mechanical and thermal energies.^[^ ^101^ ^]^ As indicated in Figure 11F–L, BaTiO_3_ (BTO), as a typical ferroelectric material, has pyroelectric and piezoelectric effects, while the ITO (indium tin oxide)‐BTO‐Ag structure can generate photovoltaic signal. The system could monitor changes in light, pressure, and temperature, but it cannot reflect these three physical parameters at the same time.

**FIGURE 12:**
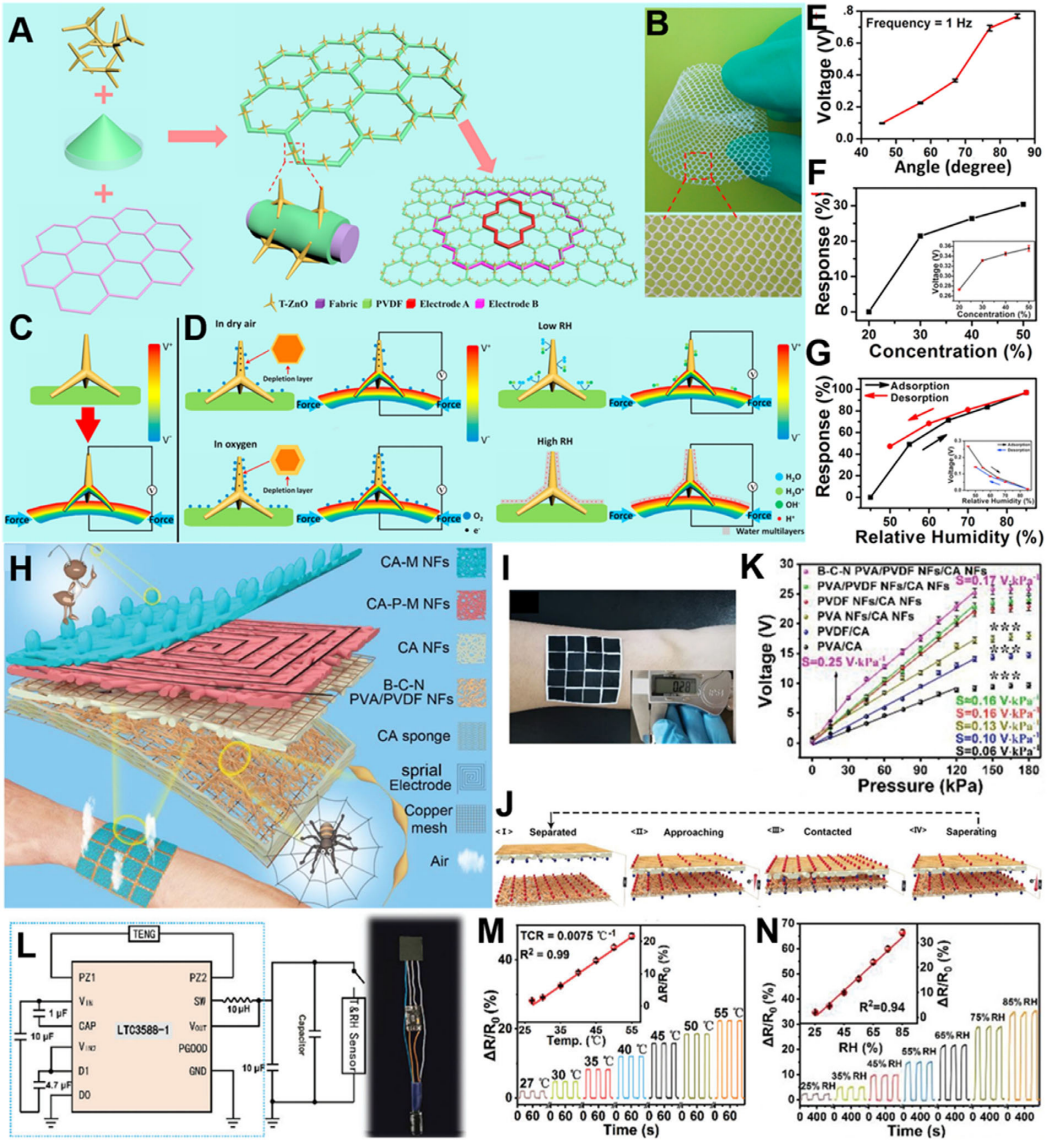
Multifunctional self‐powered e‐skin with multiple coupling effects. (A) Material selection and manufacturing process of the e‐skin. (B) Optical image of the e‐skin. (C) Piezoelectric effect for tactile perception. (D) The stress/oxygen/humidity sensing coupling effects. (E) Relationship between piezoelectric voltages and bending angles. (F) Relationship between the response variation and O_2_ concentration. The inset is the piezoelectric voltages against O_2_. (G) Relationship between the response variation and RH. The inset is the piezoelectric voltages against RH. Reproduced with permission.^[^ ^102^ ^]^ Copyright 2016, Elsevier Ltd. (H) Schematic illustration of the e‐skin. (I) Photo of the 4 × 4 e‐skin array conformally worn on human arm. The inset demonstrates that the thickness of the e‐skin is only 0.28 mm. (J) Schematic mechanism of triboelectric layer for pressure‐sensing. (K) Voltage signal and pressure sensitivity of e‐skin with different components. (L) Power management circuit (LTC3588‐1) and the integrated system circuit. (M) The response signals of temperature sensing. Inset shows the TCR values. (N) Output signals of e‐skin against RH. The inset displays the linear response to RH. Reproduced with permission.^[^ ^103^ ^]^ Copyright 2021, Wiley‐VCH

Except for the integration of energy effects, the combination of device with power generation effect and materials with specific sensing properties can also realize multifunctional sensing system. He et al. proposed a multifunctional flexible e‐skin for real‐time measuring of stress, oxygen, and relative humidity.^[^ ^102^ ^]^ Figure 12A is the structure of the e‐skin. Piezoelectric material PVDF and tetrapod ZnO (T‐ZnO) nanostructures are integrated in the fabric substrate. Figure 12B is the optical image of the e‐skin. Figure 12C,D are diagrams of the coupling principle. Stress can be sensed by the piezoelectric effect of the material under the stimulation of mechanical energy. Meanwhile, O_2_ can capture free electrons on T‐ZnO, then decrease the electron density and weaken the piezo‐screening effect, thereby enlarging the output piezoelectric signal. Water molecules will generate H_3_O^+^ ions after adsorption and act as charge carriers in H_2_O‐ZnO. These ions and free electrons in T‐ZnO have orientation migration and screen the piezoelectric polarized charges, reducing outputting performance. Therefore, the piezoelectric performance of the e‐skin will change with oxygen concentration and humidity (Figure 12E–G). This coupling effects make the e‐skin has multifunctional sensing performance. Besides, based on spider web and ant tentacle, Yue et al. construct a layer‐by‐layer multi‐sensory e‐skin, that can collect biomechanical energy via triboelectric effect and achieve simultaneous measurement of pressure, RH, and temperature.^[^ ^103^ ^]^ The schematic diagrams of the e‐skin are displayed in Figure 12H,I. The humidity‐sensing layer, temperature‐sensing layer, and triboelectric layers used for biological energy scavenging and pressure monitoring mimick organic texture and are assembled layer by layer. Figure 12J is the triboelectric mechanism of pressure sensing. From Figure 12K, this e‐skin reveals high‐pressure sensitivity of 0.25 V kPa^−1^ in 0–135 kPa. From Figure 12L, By a commercial power management circuit, the alternating current outputted by triboelectric layer is converted to direct current and provides a sustaining power supply for temperature and RH detection parts. According to Figure 12M,N, the e‐skin exhibits the temperature coefficient of resistance (TCR) of 0.0075°C^−1^ between 27 and 55°C. Furthermore, the proposed sensor system has a wide RH detection scope of 25–85% with short response and recovery time (16 and 25 s, respectively).

## 5 APPLICATION

By mimicking the comprehensive function of human skin perception, e‐skin has realized the perception of important physiological signals such as humidity, sweat, touch, and temperature, but e‐skin has some key problems restricting its further development and energy supply, so integrated e‐skin based on various self‐powered effects has become the driving force for the further progress of e‐skin. Here, self‐powered e‐skin applications in physiological health, human–computer interaction (HMI), VR, and AI are described systematically.

### 5.1 Physiological health

As technology continues to evolve, e‐skin has become an excellent perception input end. Because of the transmission of this perception, the field of e‐skin and human health can be matched perfectly. There are many sensing systems in the human body, and different parts have unique physiological signal characteristics. If the e‐skin is installed in the corresponding key parts of the body, real‐time monitoring of physiological parameters such as heart rate, breathing, blood pressure, muscle tension can be achieved for disease prevention and medical diagnosis.

**FIGURE 13:**
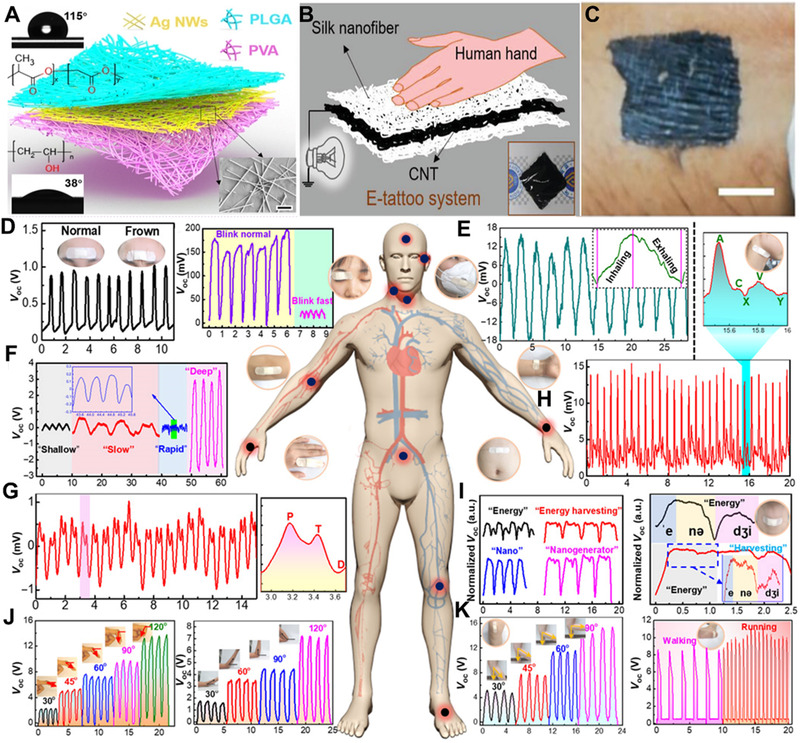
Application of self‐powered electronic skin in physiological health. (A) Structural design of e‐skin based on full‐fiber TENG. Reproduced with permission.^[^ ^104^ ^]^ Copyright 2020, American Association for the Advancement of Science. (B) Diagram of skin‐actuated E‐tattoo skin. (C) Optical image of E‐tattoo skin under stretched. (D) Voltage response to frowning and blinking movement, (E) Breathing, (F) Sleeping monitoring. (G) Pulse signals, (H) Jugular venous pulse (JVP), (I) Sound recognition, (J) Finger bending angles and the arm flexion, (K) Foot movement states. Reproduced with permission.^[^ ^105^ ^]^ Copyright 2021, Wiley‐VCH

As shown in Figure 13A, Peng et al. designed a flexible and biodegradable e‐skin based on a TENG to effectively collect movement energy and detect systemic physiological signals.^[^ ^104^ ^]^ In addition, due to the sterilization property of AgNWs, the e‐skin also has significant antibacterial property. Similarly, Gogurla et al. developed an ultra‐thin electronic tattoo using carbon nanotubes (CNTs) and silk nanofibers (SNFs), as shown in Figure 13B, which is a typical sandwich structure (SNF/CNT/SNF) and seamlessly connects to human skin (Figure 13C).^[^ ^105^ ^]^ It not only has the advantages of comfort, safety, and environmental protection but is also endowed with the functions of energy collection and high sensitivity, which can realize the monitoring of human body physiology and joint movement signals. Blinking and frowning are the most basic forms of emotional expression and the main channel for people with paralysis to express their needs. As shown in Figure 13D, these emotional changes can be successfully captured using e‐skin.

Breathing is a true reflection of a person's vital signs and plays an important role in assessing health. In order to detect respiratory flow, the electronic skin can be placed on the exhaust port of a conventional mask (Figure 13E), and the mask can reflect the breathing status of the human body in real time by changing the voltage signal. Good sleep is essential for human health, as shown in Figure 13F, where e‐skin is attached to the stomach to monitor how fast breathing occurs and judge sleep quality. Repeatable breathing patterns recorded from the abdomen have four different breathing states, including normal (light), slow, fast, and deep. Pulse is one of the key vital signs for evaluating human heart health. It can be measured by the radial artery of the wrist (Figure 13G) or the neck artery (Figure 13H).

In addition, the e‐skin has been successfully applied in speech recognition (Figure 13I) and monitoring human motion (Figure 13J–K) (such as the finger, elbow, knee, and ankle). From these demonstrations, it can be found that our e‐skin can transform various physiological features and movement behaviors into quantifiable and real‐time voltage signals, which are conducive to parallel and overall physiological and movement monitoring. Therefore, it is expected that our e‐skin will have good application prospects in the fields of disease detection and patient rehabilitation.

### 5.2 HMI

HMI is a new technology for transmitting information between humans and electronic devices, which has attracted extensive attention from researchers in recent years. The rapid development of wearable devices and robotics has put forward higher requirements for traditional interactive media, such as flexibility, portability, and low power consumption. E‐skin is a new type of HMI media that enables people to seamlessly connect with electronic devices. As a flexible sensor network, the main medium of the next generation of HMI has gradually shifted to self‐powered electronic skin due to its multiple perception capabilities.

**FIGURE 14:**
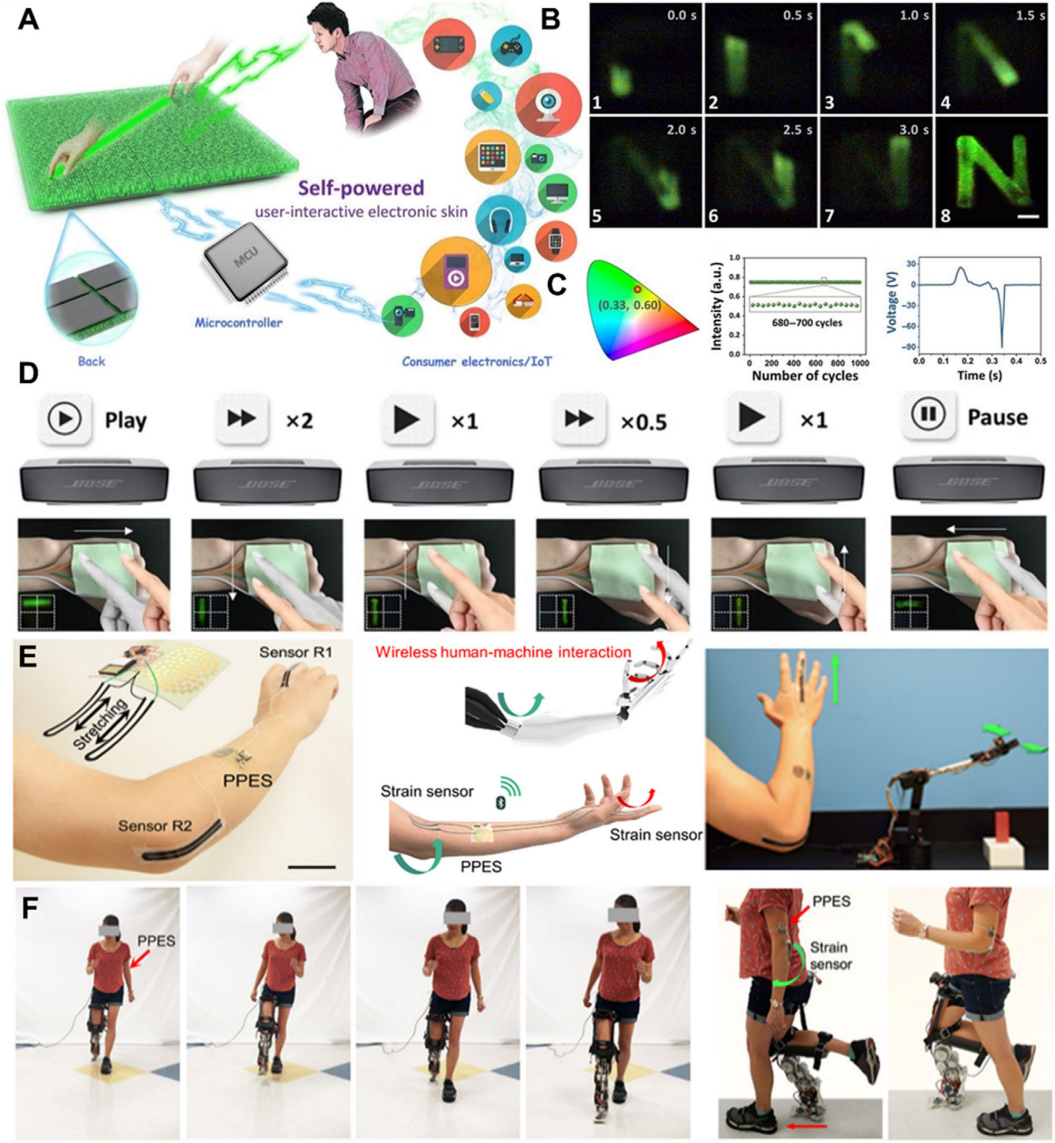
Application of self‐powered electronic skin in HMI. (A) Schematic diagram of the SUE‐skin. (B) Visual output of touch state at different times. (C) The electrical output and the chromaticity diagram of the E‐skin under sliding. (D) Touch demonstration for playing the audio. Reproduced with permission.^[^ ^106^ ^]^ Copyright 2020, American Association for the Advancement of Science. Schematic diagram of sensor recognition of (E) hand bending and (F) movement. Reproduced with permission.^[^ ^87^ ^]^ Copyright 2020, American Association for the Advancement of Science

As shown in Figure 14A, Zhao et al. prepared a simple, inexpensive, self‐powered, human–machine electronic skin based on a triboelectric‐light effect.^[^ ^106^ ^]^ Upon contact with the electronic skin, the stimulation is converted to electrical signals and instantaneous visible light (Figure 14B,C), and the device only requires a pressure of 20 kPa to trigger. On this basis, a touch operation platform can recognize more than 156 kinds of interaction logic, so as to easily control consumer electronic products (Figure 14D). The device can be used in fields such as gesture control and intelligent prosthetics. Although HMI e‐skin can project touch in space and provide visual signal output to humans, high power consumption, complex structure, and high cost are obstacles to practical application.

To expand the application of self‐powered e‐skin in the field of medical interaction, Yu et al. switched the bioelectrochemical sensor to a strain sensor and placed it on the fingers and elbows, which uses the sensor to recognize the posture of the limb and convert it into an electrical signal to manipulate the robotic arm movement grab (Figure 14E) via wireless data transmission.^[^ ^87^ ^]^ Alternatively, elbow sensors can be used to identify arm movements during walking to manipulate the mechanical prosthesis for assisted walking (Figure 14F). This e‐skin, which integrates physiological signals and molecular information monitoring, can also be used to optimize the design of future prosthetic robots, making auxiliary medical prostheses more specific and personalized.

### 5.3 VR

VR technology refers to the use of computers and other technical means to generate a realistic visual, auditory, touch, taste, and another integrated virtual environment, allowing participants to interact with objects in the virtual world through a variety of senses, thus creating an experience immersed in the real environment. However, compared with virtual hearing and vision, virtual touch often needs fast response, high‐resolution and large‐scale biocompatible devices, which makes its implementation more difficult. At present, there are two main ways to achieve virtual touch. One is through the application of mechanical force or vibration on the skin to achieve a safe and controllable virtual touch. However, this device usually requires complex and sophisticated structure design and high power consumption. Another way is to apply appropriate current on the skin surface to achieve virtual touch, which has the advantages of small size, lightweight, and high resolution.

**FIGURE 15:**
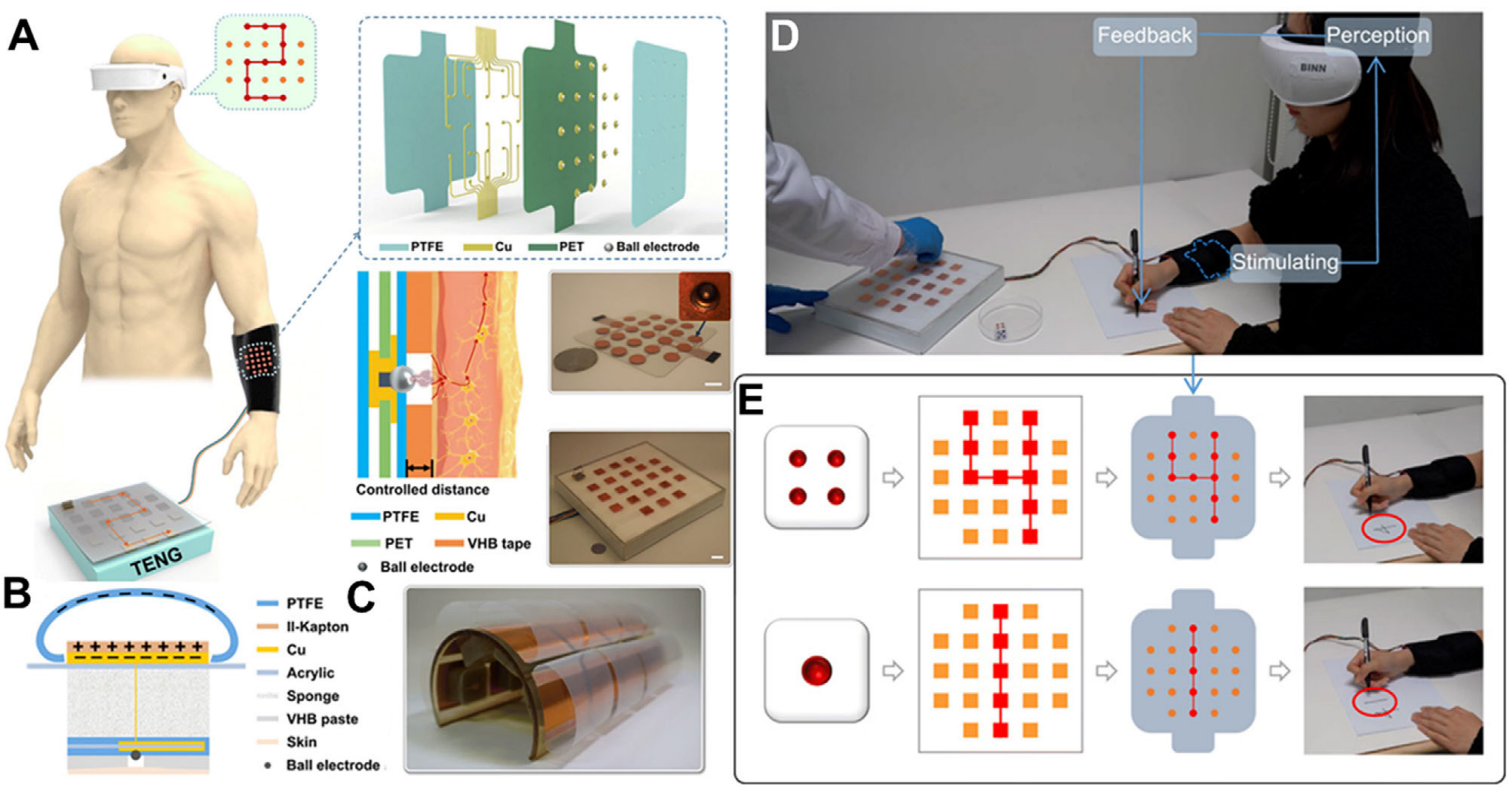
Application of self‐powered electronic skin in VR. (A)Working diagrams, structural diagrams, mechanism diagrams, and physical diagrams of virtual electro‐touch installations. (B) Unit structure diagram and (C) physical diagram of virtual electro‐touch system. (D) The ET interface tested by a girl. (E) Schematic diagram of the test. Reproduced with permission.^[^ ^107^ ^]^ Copyright 2021, American Association for the Advancement of Science

From this, Shi et al. developed a virtual electro‐touch device based on TENG and suspended electrodes array, achieving a skin‐integrated, safe, self‐powered, and painless virtual electro‐touch system.^[^ ^107^ ^]^ As shown in Figure 15A, the virtual electro‐touch system consists of TENG and a spherical electrode array integrated on the skin. The electrode array is suspended on the skin to avoid direct contact with the skin. Touch motions such as contact and friction between the system and the outside world are converted into high voltage signals by TENG. The efficient conversion efficiency of TENG drives the gap discharge between the electrodes and the skin. A high resolution, sensitive and comfortable virtual electric touch is achieved with a low current. As shown in Figure 15B,C, this virtual electro‐touch combines the effects of tactile sensing and electrostatic stimulation, in which TENG not only recognizes and senses the tactile motions input from the outside world but also converts the energy generated by touch into electrical stimulation signals that are released to the human skin.

The e‐skin sensing interface can act as a separate writing screen (Figure 15D), feeding the tactile track directly to the skin of the separated experiencer through a transmission line to achieve virtual tactile communication. As shown in Figure 15E, the author shows the application of this virtual tactile system in self‐powered braille recognition. By converting external collisions into virtual electric touch, the application of self‐powered e‐skin in VR has been broadened.

### 5.4 AI

**FIGURE 16:**
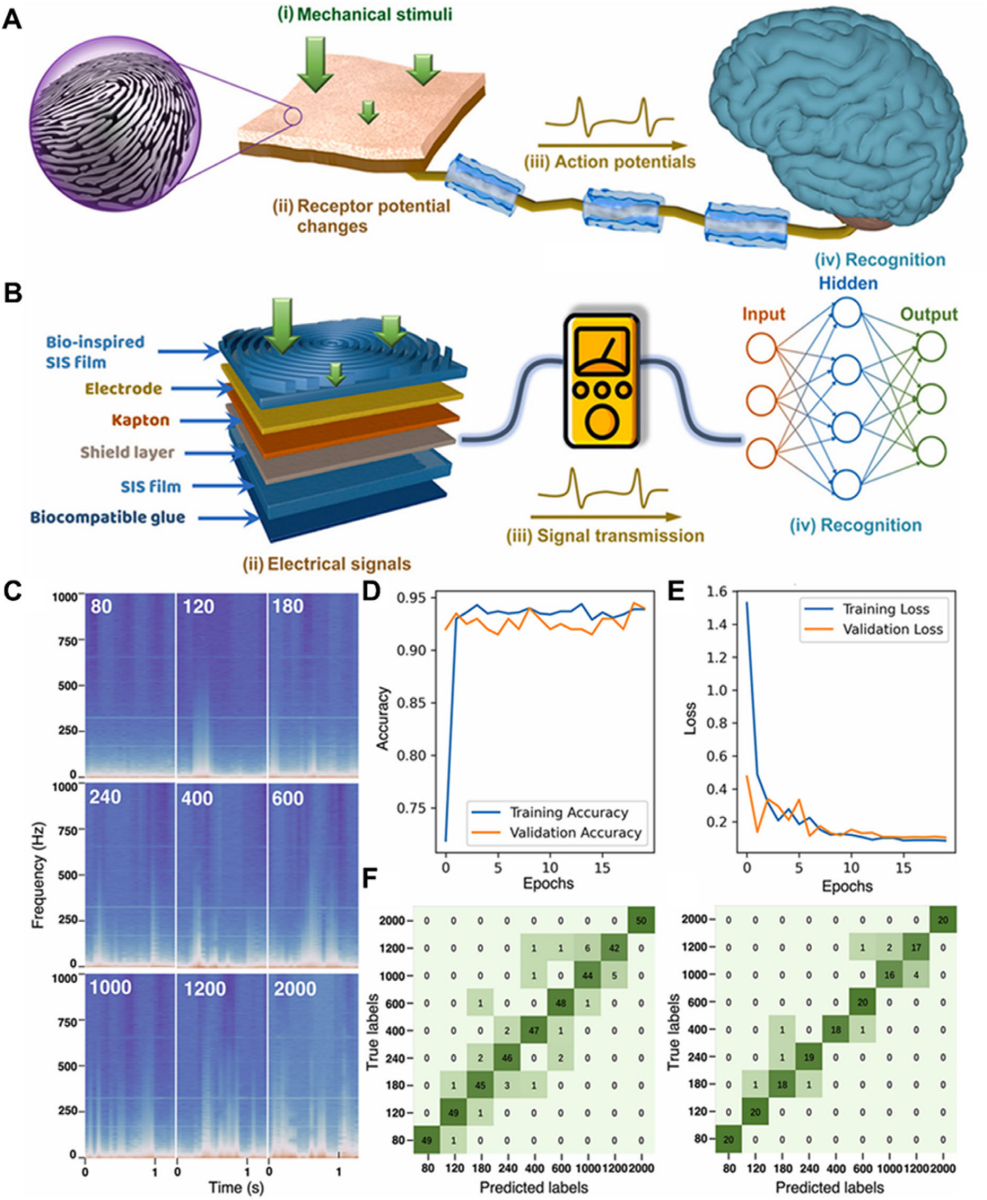
Application of self‐powered electronic skin in AI. (A) The sensing system of tactile perception. (B) Schematic diagram of bionic tactile sensing system. (C) The time‐frequency graphs of e‐skin toward different sandpapers. (D) The accuracy and (E) The loss in 20 training epochs. (F) Confusion diagram of real label and predicted label. Reproduced with permission.^[^ ^108^ ^]^ Copyright 2021, Elsevier Ltd

Self‐powered multifunctional e‐skin is highly sensitive to perception characteristics. It could generate a lot of data during the perception process. Efficient identification of useful information has become a key issue in signal processing. The introduction of AI and neural network algorithms could achieve good signal recognition functions. Zhao et al. fabricated a fingerprint heuristic electronic skin (FE‐skin) through neural network algorithms, which can effectively identify different textures by processing electrical signals collected through artificial neural networks, with a minimum texture size as low as 6.5 μm.^[^ ^108^ ^]^ In addition, the recognition accuracy of disordered and ordered textures is 93.33% and 92.5%, respectively. Figure 16A displays the specific flow of the pressure sensing system. Once the skin in the fingertips is under pressure, signals are transmitted to the brain through a protein transport network. Finally, our brain will have analysis for the information of the potential signals to determine the type, intensity, and so on of mechanical stimulation. A biomimetic tactile sensing system is designed by mimicking human beings as shown in Figure 16B. Biomimetic tactile sensing systems could collect signals and even analyze the information contained. The sensor senses the tactile information of the outside world and uses artificial neural network to process the input data, thus forming the tactile nerve. Figure 16C emphasizes the time‐frequency diagram of the e‐skin contacting different roughness sandpaper. and Figure 16D shows that the system has more than 90% accuracy over 20 training cycles. Figure 16E shows how well the predicted tags overlap with the true tags and Figure 16F shows that they are accurate and consistent. The use of artificial neural networks can greatly reduce the dependence on specific projects. The combination of reasonable sensor design and artificial neural network can accurately identify external stimuli.

## 6 CONCLUSION AND PERSPECTIVE

In this review, we compile the latest work of self‐powered multifunction e‐skin, discussing related energy sources (mechanical energy, solar energy, electromagnetic energy, thermal energy, chemical energy) and coupling techniques (double coupling and multi‐coupling). Other self‐powered electronic skin systems that have not been described in detail are summarized in Table 1. Unlike previous reviews of self‐powered sensing systems, we exclude flexible electrical energy storage with limited energy and repeatability, as well as non‐integrated power supplies, just focusing on self‐powered and self‐sensing, multifunctional e‐skin.^[^ ^120^ ^]^ Coupling effect is not only the coupling of energy sources, but also the coupling of different self‐sensing functions. Because self‐powered e‐skin has a variety of perceptual capabilities, which matches the perceptual needs of intelligent robots in the future, it can help robots perform delicate tasks, such as intelligent medical treatment, smart home, emergency rescue. In addition, the rapid development of self‐powered e‐skin is inspired by human skin. The gap between e‐skin and human skin is becoming smaller, and even has unique advantages that human skin does not have in some aspects. For example, it has the ability to monitor physiological parameters such as blood pressure, pulse, temperature, and environmental parameters (humidity and gas, etc.) in real time on top of basic tactile perception. Therefore, when the self‐powered multifunctional e‐skin is combined with brain‐computer interface (BCI), it can even act as the intelligent skin of disabled patients to perceive pain and health diagnosis in the near future. Although the potential prospect of it can be predicted, there are still many key problems difficult to solve at this stage. These relate to:

- Driving energy of multifunctional e‐skin.

**TABLE 1:** The summary of coupling effects in self‐powered multifunctional e‐skin

**Parameter numbers**	**Materials**	**Parameters**	**Mechanisms**	**Energy sources**	**Applications**	**Ref**.
One	Kapton, Cu	Pulse	Triboelectric	Mechanical energy	Disease diagnosis	^[^ ^109^ ^]^
	PANI‐PVDF	Stress	Piezoelectric	Mechanical energy	Human health monitoring	^[^ ^110^ ^]^
	GO*_x_*/Lac BC/c‐MWCNTs/AuNPs	Glucose	Redox	Chemical energy	Glucose detection	^[^ ^111^ ^]^
	MoS_2_/PU Te/PEDOT	Temperature	Thermoelectric	Heat	Wearable electronics	^[^ ^112^ ^]^
	Ag/MoO*_X_*/PTzNTz‐BOBO:PC_70_BM /ZnO/ITO	Light	Photovoltaic	Luminous energy	Medical health	^[^ ^113^ ^]^
	Ionic liquid; PDMS	Electrocardiogram	RFID	—	Human health monitoring	^[^ ^114^ ^]^
Two	PPy/PDMS	Stress; Light	Triboelectric Photo‐generated carrier	Mechanical energy Luminous energy	Brain–machine interaction	^[^ ^115^ ^]^
	LO*_x_*/Pt, PbZr*_x_* Ti_1‐_ *_x_* O_3_	Lactate; Pressure	Biofuel cell Piezoelectric	Bioenergy Mechanical energy	Human health monitoring	^[^ ^116^ ^]^
	RGO	Pressure; Temperature	Thermoelectric Finger heating effect	Heat	Wearable electronics	^[^ ^117^ ^]^
Three	BaTiO_3_; ITO; Ag	Light; Temperature; Vibration	Photovoltaic Pyroelectric Piezoelectric	Luminous energy Heat Vibration energy	—	^[^ ^118^ ^]^
Five	ZnO; Pd/ZnO, CuO/ZnO; TiO_2_/ZnO	Stress; Humidity; Ethanol; H_2_S; CH_4_	Piezoelectric	Mechanical energy	Environmental monitoring	^[^ ^119^ ^]^

The self‐powered multifunctional e‐skin mentioned in this review is mainly divided into low‐power self‐powered sensing and zero‐power self‐powered integrated system. The self‐powered integrated system can realize the wireless transmission from signal perception to signal without consuming external energy. Low‐power self‐powered sensing technology uses the harvesting energy to actively transmit the sensing signal, but the harvesting energy is small, and the back‐end circuit is still needed to process and transmit the signal. Therefore, improving the power of energy and developing the corresponding low‐power back‐end circuit could improve the practicability of self‐powered multifunctional e‐skin.

II. Multidimensional perception of e‐skin.

The ideal e‐skin expects to obtain the perception ability that is not inferior to that of human skin, but it will bring significant interference when realizing a variety of perception abilities. Integrating a variety of energy sensing effects into one device and solving the interference problem through ingenious structure design can further develop the self‐powered e‐skin.

III. Material choice of self‐powered multifunctional e‐skin.

E‐skin attached to human body or robot has good flexibility. Long time contact with human body requires it to have good biocompatibility and air permeability. In addition, due to the aging wear and accidental damage in the face of long‐term use, the new generation of e‐skin should have self‐healing performance.^[^ ^121^ ^]^

IV. Back end circuit of self‐powered multifunctional e‐skin.

For the integrated system that integrates the back‐end circuit into the e‐skin, the flexible back‐end circuit design is the key to maintain the flexibility of the whole system. In the future, printed circuit technology will be used to realize flexible printed circuit to replace the current rigid print circuit board. In addition, in the case of limited driving power, low‐power signal processing, and transmission circuit is meaningful. In the design of low‐power signal processing and transmission circuit, lower power supply voltage, slower clock frequency, and smaller distributed capacitance are selected to effectively reduce the circuit power consumption.

## CONFLICT OF INTEREST

There are no conflicts to declare.

## AUTHOR CONTRIBUTIONS

Xuhui Sun, Zhen Wen, and Yunfeng Chen conceived ideas and structures and prepared the manuscript. Zhengqiu Gao and Fangjia Zhang organized references, performed tables and figures.
